# The Influence of Miscibility of Some PLA-Based Bio-Hybrids Designed for 3D Printing and Medium-Life Applications on Their Physical Aging and Thermodynamic Stability

**DOI:** 10.3390/polym18010061

**Published:** 2025-12-25

**Authors:** Doina Dimonie, Silvia Mathe, Roxana Doina Trușcă, Celina Maria Damian, Maria Râpă, Ștefan-Ovidiu Dima, Ștefan Dumitru, Florin Oancea

**Affiliations:** 1 Faculty of Chemical Engineering and Biotechnologies-Doctoral School, National University of Science and Technology POLITEHNICA, 011061 Bucharest, Romania; 2 National Institute for Research and Development in Chemistry and Petrochemistry, 060021 Bucharest, Romania; 3 National Research Centre for Micro and Nanomaterials, Faculty of Chemical Engineering and Biotechnology, National University of Science and Technology POLITEHNICA, 060042 Bucharest, Romania; 4 Advanced Polymer Materials Group, National University of Science and Technology POLITEHNICA, 011061 Bucharest, Romania; 5 Department of Metallic Materials Processing and Environment Engineering, Faculty of Material Science and Engineering, National University of Science and Technology POLITEHNICA, 060042 Bucharest, Romania; 6 Department of Mechanics, National University of Science and Technology POLITEHNICA, 060042 Bucharest, Romania

**Keywords:** PLA, bio-hybrids, compatibility, miscibility, time behavior, thermodynamic stability, 3D printing, medium lifetime

## Abstract

The aim of this article was to identify the dependence of the physical aging of PLA-talc-PCL bio-hybrids, with or without a nucleating agent (NA), produced by melt compounding and designed for 3D printing for medium-life applications, on the degree of miscibility, and to identify a formulation with a heat deformation temperature (HDT) of practical interest and thermodynamical stability for at least two years. The obtained bio-hybrids were characterized to illustrate their miscibility and long-term thermodynamic stability, both initially and after two years. The preservation of properties over time was analyzed by examining the observed physical aging, since its can be associated with changes incompatible with 3D-printed items used as structural automative components. Without using NA, two partially miscible bio-hybrids with multiphase, polymorphic morphology were achieved, which showed strong physical aging and thermodynamic instability over two years. The use of NA led to a bio-hybrid with a relatively narrow single melting peak, which showed good miscibility, without physical aging or thermodynamic instability over the two-year period. The morpho-structural and functional characterization of the selected formulation will be further investigated and possibly corrected, to advance to the next level of scale-up.

## 1. Introduction

Polylactic acid (PLA) is a renewable, biocompatible polymer [1], compostable under controlled conditions [2], with good mechanical properties; however, it is brittle [3]. PLA is light and transparent [1], versatile in terms of processing into finished items, recyclable [4], but has low thermal stability. It is used mainly in the food, textile, and medical industries [5]. To widen its application areas by controlling functional properties, methods such as physical modification (with shock modifiers, fillers, and/or plasticizer) [6] and reactive extrusion [7]) are employed. These approaches can increase molecular weights and control polydispersity [8], leading to complex compounds and bio-nano-hybrids [9] for applications in medicine, food, and electronics [10]. In the automotive industry, PLA can be used only for interior components (dashboards, door panels, and air filters [11]) and not for exterior parts. However, many known attempts have been made to upgrade PLA properties and processing techniques, such as 3D printing [12,13].

The durability of polymeric materials is defined as the ability to withstand physical (weathering, extreme temperatures) and/or chemical attacks without morpho-structural changes (cracking, oxidation, wear, etc.) and while maintaining functional performance throughout the designed lifetime [14,15,16,17,18]. At first sight, PLA durability appears indefinite if it is not affected by destructive factors. However, PLA durability can be disturbed by physical aging, which potentiates chemical degradation. Physical aging of the amorphous polymers is related to the non-thermodynamic equilibrium state of the macromolecules and their tendency to reach, through relaxation, a more thermodynamic stable configuration [19,20]. The smaller the difference between the two energetic levels, the faster the rearrangement of macromolecules from the amorphous phase into a more thermodynamically stable state [21,22]. The scale of these rearrangements generates material densification, a decrease in macromolecular mobility and compounds’ ductility [23], an increase in glass transition temperature [24], and increases in crystallinity, enthalpy, and density [25,26]. These changes generate brittleness and rigidity [27] and ultimately lead to physical destruction by cracking and breaking [28,29], contributing to microplastic formation. This process is extremely dangerous, especially for large plastic items [30,31], because, it can also cause phase separation, plasticizer leaching, and a decrease in mechanical properties [32,33,34,35]. Physical aging of semi-crystalline polymers can be generated by defects in the contained crystals (chemical impurities, misalignment of crystal planes, dislocations, chain-end defects, branching, tacticity disorders, micro-pores, or cracks) [36], which can destabilize the crystal structure as a whole and generate secondary crystallization, leading to smaller and more stable crystals. Depending on crystallization conditions and polymer type, physical aging decrease or increase crystallinity through well-known mechanisms [37,38,39]. Physical aging should not be confused with chemical aging, which it can potentiate [19,20] and which involves reactions that break covalent bonds [19,20,40]. All these changes make polymer compounds incompatible with durable applications.

In addition to other properties that need to be corrected, PLA is affected by physical aging even at room temperature [41], which enhances the chemical degradation and makes it unsuitable for medium-life applications.

Modifying PLA with talc is a commonly used method to increase stiffness, improve melt processability [42], and enhance thermal and mechanical properties [43]. Depending on its quantity and type, talc can act as nucleating and reinforcing agent [44,45]. Since PLA is brittle, in order to increase its ductility and ability to store deformation energy, it can be modified with poly-ε-caprolactone (PCL) at contents no more than 30% [46,47], and also with certain plasticizers [48,49]. The incorporation of inorganic fillers is more efficient when PLA macromolecules are more relaxed, a state favored by the presence of plasticizers [50], which decrease the glass transition temperature and increase toughness [51]. PCL, a biodegradable polyester, can be used as an additive [52] with a plasticizing function [53] and is often employed in bio-polymers with self-healing properties or drug delivery systems [54]. Depending on its quantity, PLA/PCL compounds are partially compatible, and the presence of PCL also increases composting speed [55]. Melt compounding and 3D printing melt-processing methods require compounds with narrow melting temperature ranges, easily controllable processing behavior and stable functional properties over time at the performance level required by the intended application (e.g., the automotive industry).

The aim of this article was to identify the dependence of physical aging in PLA-talc-PCL bio-hybrids, with or without a nucleating agent, made from melt compounding and designed for 3D printing for medium-life applications, on the degree of miscibility, and to identify formulations thermodynamically stable with heat deflection temperatures (HDTs) of practical interest.

## 2. Materials and Methods

### 2.1. Procedure

To obtain 3D-printable bio-hybrids for medium-life applications, PLA was physically modified by melt compounding with talc and PCL as an interfacial agent, chosen also to avoid the use of liquid plasticizers, which can migrate over time and thus causing physical aging of the bio-hybrids. To control the morphology and the obtained stability, in certain formulations a fourth component, namely a nucleating agent, was used. Except for the nucleating agent, all other three components were dried before melt compounding. The obtained bio-hybrids were then kept for more than two years under laboratory conditions at a temperature of 25 °C +/− 3 °C. The period of 2.2 years represents the time after which the first bio-hybrid breakage appeared. The new bio-hybrids were morpho-structurally and functionally characterized, both initially and after 2.2 years, through methods that highlight miscibility, time stability, and thus possible physical aging as an expression of thermodynamic stability. Testing was continued for the bio-hybrids that did not change significantly after more than two years, extending up to four years. The bio-hybrids with unchanged or no significant changes were selected for in-depth morpho-structural and functional characterization and, subsequently, for formulation corrections aimed at scaling up and 3D printing.

### 2.2. Materials (S1)

The used materials were as follows: PLA (M_w_ = 11.6 × 10^4^ g/mol; dextro content: 5%, MFR: 14 g/10 min, T_m_ = 145–160 °C; density: 1.24 g/cm^3^, HDT 55 °C (S1_Neat PLA)), talc (Mg_3_Si_4_O_10_ (OH)_2_, particles of 2 nm–16.33 µm, density: 2.7 g/cm^3^ (S1_Talc)), PCL (M_w_ = 600 g/mol; T_m_ = 58–60 °C; density 1.08–1.12 g/cm^3^ (S1_PCL)), and a nucleating agent (potassium 3,5-bis (methoxy carbonyl) benzene sulfonate-LAK 301, Teknor Apex, Pawtucket, RI, USA (S1_Lak 301)).

### 2.3. Bio-Hybrids Preparation

PLA was dried at 80 °C, PCL at 40 °C, and talc at 100 °C in vacuum ovens for 4 h/6 h/2 h, respectively. Compounds with the following compositions were prepared: (100) [p] PLA, (15–75) [p] talc, (0–30) [p] PCL, and (0–6 [p]) nucleating agent (Table S2.1).The bio-hybrids were obtained by melt compounding, at selected blending ratios, in a Brabender mixer using a classical working sequence (190–200 °C, 100 rpm), followed by roll profiling (50–75 °C, 25 rpm_1_, 25 rpm_2_) to produce sheets of 0.5 ± 0.05 mm thickness. The sheets were subsequently compression molded (210–220 °C platen temperature, 5–7 min of preheating time, 10–15 min under pressure, 15–20 min of cooling time, pressure 200), yielding plates with dimensions of 80 × 40 × 4 mm,1.201 g/cm^3^–1.532 g/cm^3^ for each pressing. 

### 2.4. Characterization

#### 2.4.1. Chemical Structure 

It was studied through FTIR-ATR (Spectrum 100, Perkin Elmer, Shelton, CT, USA) equipped with a diamond, from 4000 cm^−1^ to 600 cm^−1^, with 32 scans/bio-hybrid.

#### 2.4.2. Morphological Structure 

It was analyzed by X-ray diffraction (XRD) (D8 discover diffractometer, Bruker AXS GmbH, Karlsruhe, Germany) using working radiation CuKα1, λ = 0.15406 nm, radiant incident X-ray diffraction (GIXRD) over a range of 3°–100°, and by Surface Electron Microscopy (SEM) (Vega XMU microscope, TESCAN, Brno, Czech Republic) on fractured gold-coated samples.

#### 2.4.3. Thermal Behavior

Differential Scanning Calorimetry (DSC) (DSC3, Mettler Toledo, Greifensee, Switzerland) was performed using the following procedure. To eliminate thermal history, samples were subjected to first heating from 20 °C to 200 °C (10 °C/min), cooling from 200 °C to 20 °C (2 °C/min), second heating from 20 °C to 250 °C (10 °C/min), with a 2 min isotherm between runs. DSC analysis was focused on the positive temperature range relevant to future 3D printing of selected compounds for automative items. The following thermal values were measured: glass transition (Tg), melting (Tm) and crystalization (Tc) temperatures, melting and crystallization enthalpy, and crystallinity (X) (1), where ∆Hm: melting enthalpy, ∆Hcc: cold crystallization enthalpy, ∆H°m: melting enthalpy of a 100% crystalline PLA (93.1 J/g), and wPLA-PLA weight fraction in the bio-hybrid [56,57]

(1) $$\%X=\frac{\Delta Hm-\Delta Hcc}{\Delta H^{{}^{\circ}}m\cdot w_{PLA}}\cdot 100.$$

#### 2.4.4. Physical Properties

Density (Density balance XP, Mettler Toledo, Greifensee, Switzerland): ISO 1183 [58], density balance, distilled water immersion method.

Shore A hardness (Shore A Durometer, ZwickRoell, Ulm, Germany): ISO 48 [59] from 2018.

#### 2.4.5. Functional Properties

Heat deflection temperature (HDT) (DMA Q800, TA Instruments, Leatherhead, UK), equipment with three-point bending clamp, 50.00 × 12.23 × 3.00 mm specimens, heated from 20 °C to 175 °C (2 °C/min), ISO 75 [60]. Mechanical behavior: qualitative estimation, from time to time of impact (hitting the bent material with a 5 kg weight) and flexural strength (50 successive folds and unfolds at room temperature).

#### 2.4.6. Physical Aging

Physical aging was evaluated through time variation in chemical–morphological structure and in functional properties (thermal behavior, physical and mechanical properties, (density, durability), and heat deflection temperature (HDT)), both initially and after 2.2 years.

## 3. Results

In semi-crystalline polymers, the intensity of FTIR peaks is directly related to the degree of crystallinity and the precision of macromolecular arrangement within the crystals. It is also known that strong interactions indicating component compatibility reduce the degree of crystallization order, generating lower peak intensities [61]. Comparative analysis of the initial and 2.2-year FTIR spectra, considering the common significant low-intensity peaks (Figure S3.1), allowed selection of bio-hybrids based on their degree of compatibility. In addition, it was observed that formulations with small PCL quantity (0.6–0.8%), regardless of talc amount (13–42%), did not maintain their properties over time, whereas bio-hybrids with higher contents (4–21%) did not lose their properties for at least 2.2 years (Table S2.1). Therefore, two such bio-hybrids were analyzed: one with a reduced PCL amount of 3.5% and another with 16% PCL.

### 3.1. Bio-Hybrids with 3.5% PCL (Talc: 27.5%/(RT 93))

#### 3.1.1. Compatibility and Miscibility

**Figure 1:**
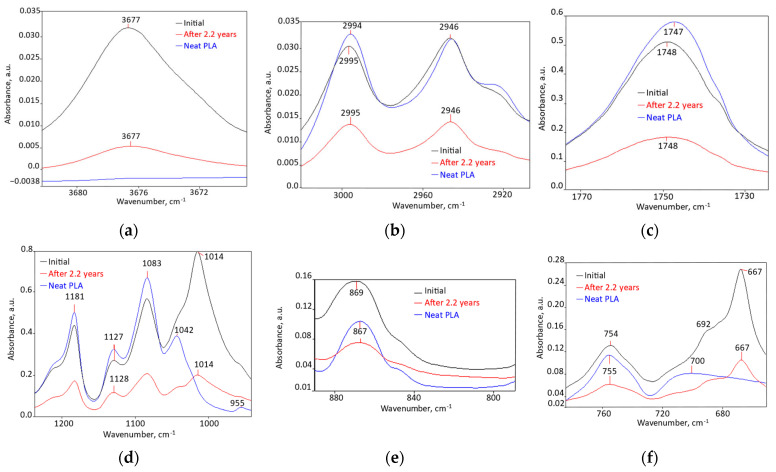
FTIR spectrum of bio-hybrid with 3.5% PCL (RT 93), initially and after 2.2 years compared with those of PLA, in the following spectral ranges: (**a**) 3684 cm^−1^–3668 cm^−1^; (**b**) 3020 cm^−1^–2900 cm^−1^; (**c**) 1800 cm^−1^–1700 cm^−1^; (**d**) 1230 cm^−1^–940 cm^−1^; (**e**) 900 cm^−1^–780 cm^−1^; (**f**) 800 cm^−1^–620 cm^−1^.

Chemical structure (Figure 1, Tables S1.1, S1.5, S1.7, S1.9, S2.1 and S3.3 [62,63,64,65,66,67,68]): components miscibility was proved by the following changes: disappearance of individual component peaks, peaks shift greater than 10 cm^−1^, widening absorptions, and intensity reductions by more than 50% relative to the matrix polymer peaks. Minor spectral changes could not be used in this estimation [69,70,71,72]. A description of all changes induced by melt compounding, in relation to PLA and the other two components, both initially and after 2.2 years, are presented in detail in Tables S3.1–S3.3.

These spectra reveal that, as a result of melt compounding, 47 changes occurred, of which 42 of them denote some degree of miscibility: a newly appeared peak; 29 (61%) disappeared peaks, 11 from PLA (52%), 15 from PCL (79%), and 3 from talc (43%); 13 peaks shifted by more than 10 cm^−1^ (28%) (10 peaks from PLA (48%)); and 7 peaks have intensity modified by more than 50%. Practically all PLA peaks have undergone changes indicating at least interfacial compatibility, and even partial miscibility. Of the 19 PCL peaks, only 2 (10%) did not suffer modifications compatible with miscibility, and for talc only one peak (14% of the total) was not modified.

**Figure 2:**
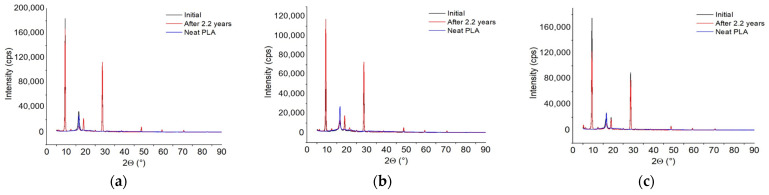
Morphological structure of the bio-hybrid with (**a**) 3.5% PCL; (**b**) 16% PCL; (**c**) 3.5% PCL and 3% nucleating agent.

The morphology (Figure 2 and Figures S1.2, S1.4, S1.6 and S1.7 and Tables S1.4, S1.6, S1.8 and S1.10) is multiphasic (crystallinity 29.7%) and polymorphic, characterized by six diffraction peaks, compared with a total of 30 peaks from the 3 components (Table S4.1). Two main peaks were observed: one at 2θ of 9°, most likely originating from talc but with higher intensity (180,000 counts/s in the bio-hybrid compared with 900 counts/s in talc) and another at 2θ of 29°, also originating from talc, with higher intensity (110,000 counts/s compared with 15,000 counts/s). The other 15 diffractions of talc, all 8 diffractions of PCL, and 5 diffractions of PLA no longer appear in the bio-hybrid. These results seem to demonstrate a morphological arrangement in which crystallinity is formed by PLA chains around talc particles, probably the smaller ones, suggesting a nucleating effect. It is also possible that the larger particles act as a reinforcing filler.

**Figure 3:**
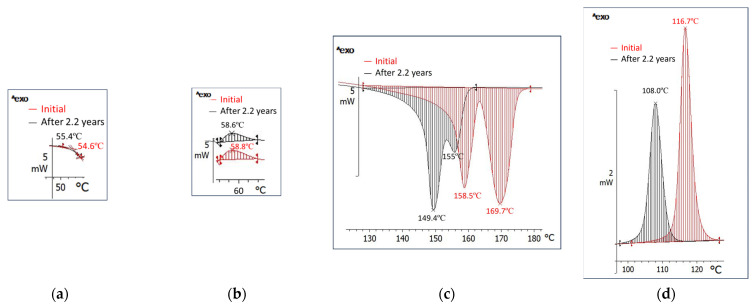
Glass transition temperature (**a**), cold crystallization (**b**), melting (**c**), and crystallization (**d**) of the bio-hybrid with 3.5% PCL (RT 93).

Comparing the thermal behavior (Figure 3 and Figures S1.1, S1.8 and S1.11 and Table 1 and Table 2, and Tables S1.2, S1.3 and S1.11) of the bio-hybrid with that of the matrix polymer, a slight decrease in the glass transition temperature from 60.0 °C to 54.6 °C (Figure 3a) and cold crystallization can be observed. For PLA, cold crystallization occurs between 101 °C and 145 °C with an enthalpy variation of 0.7 J/g, whereas for the bio-hybrid it occurs between 34 °C and 64 °C with an enthalpy variation of 0.5 J/g. While PLA melting is monomodal, occurring between 145 °C and 165 °C, with a maximum at 153.2 °C and an enthalpy of 0.77 J/g, the bio-hybrid melting is bimodal (Figure 3c), occurs between 128 °C and 179 °C with maxima at 158.5 °C and 167.7 °C, and has an enthalpy of 0.5 J/g (Table 1). The bio-hybrid crystallizes at cooling between 127 °C and 101 °C with a maximum at 116.7 °C and it has 29.7% crystallinity, whereas PLA has only 0.08% crystallinity, making crystallization almost imperceptible.

First, the bio-hybrid exhibits bimodal melting over a wide range above 100 °C (130–175 °C), unlike PCL, which melts below 100 °C (10 °C to 80 °C, with a maximum at 62 °C-S1_Neat PCL), and somewhat closer to PLA (monomodally, 145–165 °C with a maximum at 153.2 °C, S1_Neat PLA). Cold crystallization is still preserved. These melting characteristics of the bio-hybrid prove that, as it was seen in the FTIR analysis, the interfacial changes caused by PCL are limited.

The SEM (Figure S5.1) shows semi-crystalline, inhomogeneous morphology, with flow fronts totally different from the matrix polymer, quite ordered in certain directions.

**Figure 4:**
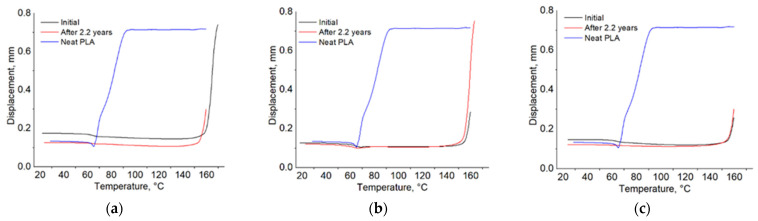
HDT for bio-hybrids RT 93 (**a**), 108 (**b**), 103 (**c**).

HDT is almost 2.5 times higher than that of unmodified PLA, i.e., 150 °C versus 67 °C (Figure 4a).

The density is 1.355 g/cm^3^ and the hardness is 95°Sh A (Table S6.1). The material is resistant.

#### 3.1.2. Time Behavior: Thermodynamic Stability

Compared with the initial bio-hybrid properties, after 2.2 years the following changes were observed:

Chemical Structure (Figure 1, Table S3.3): all the 15 bio-hybrid FTIR peaks were found in the spectrum, but they had (56.25–64.29%) lower intensities, which indicates substantial changes.

Morphological Structure (Figure 2a, Table S4.1): the number of diffraction peaks increased due to the appearance of peaks belonging to the three components, and the formation of new low-intensity peaks at 2θ of 55°, 65°, and 73°, means increased polymorphism. Crystallinity decreased by 6.3%, from 29.7% to 23.4% (Table 2).

Thermal behavior (Figure 3, Table 1 and Table 2) results confirm the morphological rearrangement highlighted by XRD results and the 6.3% decrease in crystallinity. Tg is almost unchanged, and cold crystallization occurs practically in the same melting range with the same maximum (Table 1). Melting takes place over an interval shifted 34 °C to lower temperatures and it is also bimodal, but the second peak appears as a shoulder. The melting maxima are also recorded at lower temperatures by 8.6 °C and 14.7 °C (shoulder), and the needed enthalpy decreases by 5.8 J/g. This demonstrates that smaller crystals appeared over 2.2 years and melt at lower temperatures. Crystallization shifts by 8.7 °C to lower temperatures, demonstrating smaller crystals formation. The crystallization enthalpy decreases by 5.2 J/g as a consequence of reduced crystallinity.

The HDT decreases by 10 °C.

Density increases by 0.056 g/cm^3^, while hardness remains almost unchanged (Table S6.1).

SEM morphology: although more organized, strong inhomogeneity remains (Figure S5.1).

Mechanical properties seem to be unaffected by the changes described (Table S7.1).

The results described above prove that the bio-hybrid with 3.5% PCL presents partial compatibility and possible partial miscibility. Over 2.2 years, its macromolecules tend toward a thermodynamic equilibrium state with the lowest stored energy, a process denoted as physical aging, which occurs during this period.

### 3.2. Bio-Hybrids with 16% PCL (Talc:20%; RT 108)

#### 3.2.1. Compatibility, Miscibility

**Figure 5:**
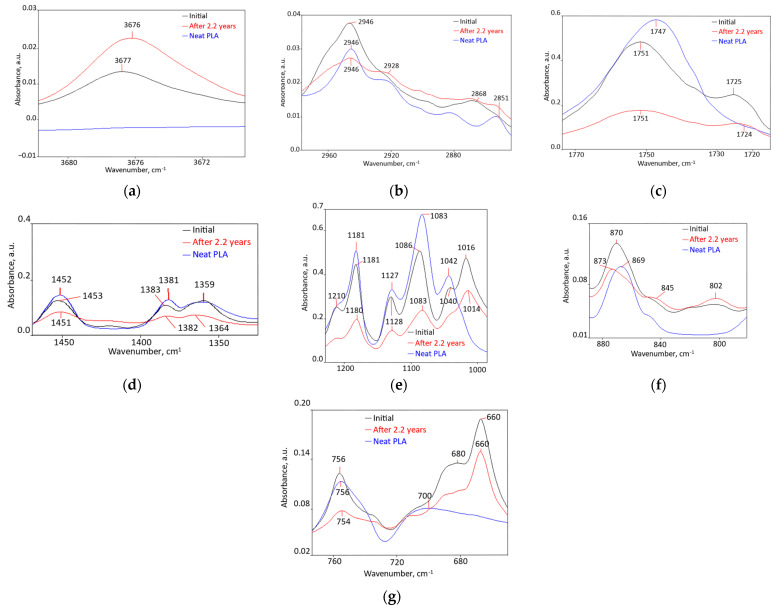
FTIR chemical structure of the RT 108 bio-hybrid, initially and after 2.2 years compared with that of the PLA matrix, in different spectral ranges: (**a**) 3684 cm^−1^–3668 cm^−1^; (**b**) 2980 cm^−1^–2840 cm^−1^; (**c**) 1770 cm^−1^–1710 cm^−1^; (**d**) 1470 cm^−1^–1320 cm^−1^; (**e**) 1230 cm^−1^–970 cm^−1^; (**f**) 900 cm^−1^–780 cm^−1^; (**g**) 780 cm^−1^–650 cm^−1^.

Chemical Structure (Figure 5, Tables S1.1, S1.5, S1.7, S1.9 and S3.6, [62,73,74,75,76,77,78,79]): of the 47 FTIR absorption peaks of the components, 20 (43%) disappeared (7 from PLA (33%)), 11 from PCL (67%), and 2 from talc (29%). Seven peaks (15%) shifted by more than 10 cm^−1^, and the intensity of eight peaks (17%) was modified by more than 50%. These 35 FTIR modifications (74%) are compatible with miscibility, which means that the components from this bio-hybrid are partially compatible and possibly partially miscible.

Morphology (Figure 4b, Table S4.1): with one exception, the diffraction peaks of the bio-hybrid occur at angles distinctive of the components. Among the nine diffraction peaks of the bio-hybrid, only those from 2θ of 22° come from PLA, but with much lower intensity (18,000 counts/s in the bio-hybrid and 35,000 counts/s in PLA). Otherwise, the bio-hybrid diffractions intensities are generally lower, except those at 13° of 55,000 counts/s, which is somewhat similar to those from talc at 2θ of 9° with an intensity of 55,000 counts/s. The morphology is multiphase (5% crystallinity) with polymorphic crystals.

**Figure 6:**
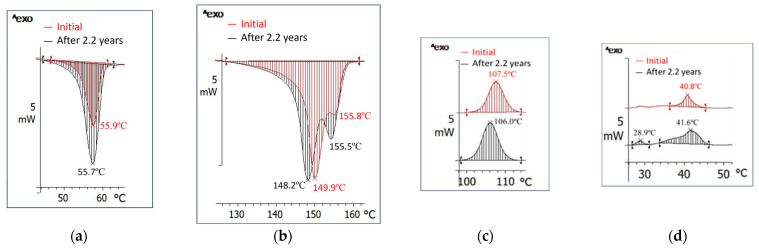
Melting (**a**,**b**) and crystallization (**c**,**d**) of the bio-hybrid with 16% PCL (RT 108).

Thermal Behavior (Figure 6, Table 3): this bio-hybrid does not have a glass transition in the positive temperature range, likely due to its high PCL content with Tg at −60 °C. It melts by consuming 3.6 J/g (Table 3) in two ranges: one below 100 °C, monomodal, between 47 °C and 61 °C with a maximum at 55.9 °C, consuming 1 J/g (Figure 6a, Table 3), and almost near to PCL melting; and a second range above 100 °C, bimodal, between 127 °C and 162 °C with maxima at 149.9 °C and 155.8 °C, and very close to PLA melting. Crystallization also occurs in two ranges: one up to 100 °C, between 115 °C and 100 °C, releasing 3.2 J/g, and the second one between 46 °C and 41 °C, with a max at 40.8 °C. The crystals are smaller and have variable sizes.

The SEM morphology (Figures S1.3, S1.5 and S5.2) shows that starting from the block-type morphology with discontinuous fracture fronts of the PLA matrix (Figure S5.2), modification according to this formulation led to a morphology with homogeneous fracture fronts, oriented in the flow direction.

The HDT (Figure 4b) is 153 °C compared with 68 °C for neat PLA.

Density is 1.289 g/cm^3^ while hardness is 91°Sh A. The material is resistant (Table S7.1).

#### 3.2.2. Time Behavior: Thermodynamic Stability

Relative to the initial bio-hybrid, after 2.2 years the following changes occurred:

Chemical structure (Figure 5, Table S3.6): two peaks disappeared, the intensities of fifteen peaks decreased by 12.5–68.75%, whereas for the six other peaks the intensities increased by 4–76.92%.

XRD morphology (Figure 4b and Table S4.1): the crystallinity increased by 1.8%, up to 6.8%, and the diffraction peaks increased to 11. Three diffractions occur at 2θ values identical to those of the initial bio-hybrid but have higher intensities. Additionally, two new diffractions appear at 2θ of 11° and 64°, which do not belong to the components. The diffraction from 2θ of 30° is not found in the initial bio-hybrid but comes from PCL. Because of all these changes, the crystallinity has increased slightly. The multiphasic morphology is also polymorphic because the new appeared crystals are inhomogeneous in size.

DSC behavior shows that the first melting range widens by 19 °C and becomes 44–63 °C, with a maximum at 55.7 °C, a value almost identical to the first melting maximum of the initial bio-hybrid, but needing a slightly higher fusion heat of 1.9 J/g towards 1 J/g, which means a slight increase in crystallinity and formation of small crystals with a wide size distribution. The second melting occurs over a 35 °C range, between 127 °C and 162 °C, it is also bimodal, has maxima at 148.2 °C and 155.5 °C, and needs a higher enthalpy of 4.5 J/g vs. 3.6 J/g for the initial bio-hybrid. Crystallization also occurs in two stages.

The SEM morphology (Figure S5.2) is still inhomogeneous, with wider and slightly fractured fronts. Density is almost unchanged, and hardness (Table S6.1) does not change.

These results prove that this bio-hybrid exhibits limited compatibility; and over 2.2 years it undergoes many changes because it tried to find the thermodynamic equilibrium with the lowest stored energy within a physical aging process.

### 3.3. Bio-Hybrids with 3.5% PCL and 3% Nucleating Agent (Talc:40%, (RT 103))

#### 3.3.1. Compatibility, Miscibility

**Figure 7:**
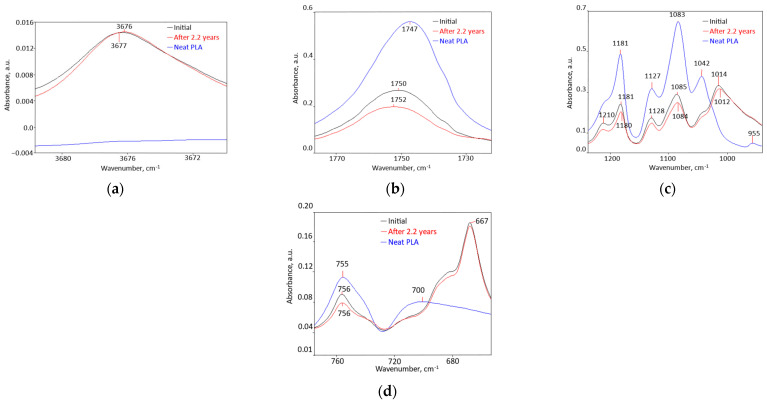
FTIR spectra of the bio-hybrid with 3.5% PCL and 3% nucleation agent (RT 103), initial and after 2.2 years compared with those of the matrix PLA, in different spectral ranges: (**a**) 3682 cm^−1^–3670 cm^−1^; (**b**) 1760 cm^−1^−1720 cm^−1^; (**c**) 1230 cm^−1^–940 cm^−1^; (**d**) 770 cm^−1^–650 cm^−1^.

Chemical Structure (Figure 7 and Tables S3.7–S3.9): the ten peaks of the bio-hybrid mainly result from interactions between PLA (matrix) and the other three components, in combinations with one or two of them. The only bio-hybrid peak that belongs to a component is the one at 3676 cm^−1^, which most probably comes from talc. The peak at 1014 cm^−1^ is not found in any of the components. These results seem to reveal improved compatibility and possibly miscibility among the components of this bio-hybrid.

XRD Morphology (Figure 4c and Figures S1.2, S1.4, S1.6–S1.8 and S1.10 and Tables S1.4 and S4.1): of the 35 different diffraction peaks of the components, only 4 appear in the bio-hybrid, 3 at angles different from those of the components, and 1 of them identical to that of PLA at 2θ of 23°. This means that during melt compounding, the components lost their integrity and generated a new morphology with 19% crystallinity, containing polymorphic crystals.

**Figure 8:**
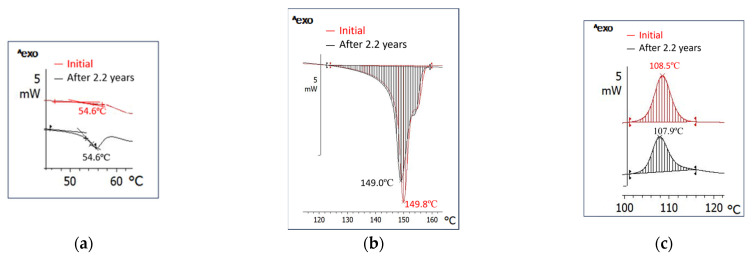
Glass transition (**a**), melting (**b**), and crystallization (**c**), initially and after 2.2 years of the bio-hybrid with 16% PCL (RT 103).

Thermal Behavior (Figure 8 and Figures S1.1, S1.2 and S1.8, Table 4 and Tables S1.2, S1.3 and S1.11): if unmodified PLA has a Tg of 61.7 °C and a crystallinity of 0.8%, the compound has a Tg of 54.6 °C and a 19.3% crystalline phase that melts monomodally, in a much narrower range from 130 °C to 159 °C, with a peak at 149.8 °C and a small shoulder at 154 °C, and needs 17.9 J/g for melting (Figure 8). Crystallization is also monomodal and occurs in a narrower range of 15 °C, between 116 °C and 101 °C, with a maximum at 108.5 °C and release of 15.8 J/g. It is noted that the disappearance of PCL and PLA melting peaks is proof of advanced compatibility, and even miscibility, among the components of this bio-hybrid.

SEM Morphology (Figure S5.3): the structure is inhomogeneous and has a mixed appearance, with fracture fronts that are not aligned with the flow direction, together with narrow, continuous areas of compact structure, differentiated from the fronts with which they coexist.

The HDT (Figure 4c) is 145 °C compared with 65 °C for unmodified PLA.

The density is 1.510 g/cm^3^ and the hardness 95°Sh A. The material is resistant.

#### 3.3.2. Time Behavior: Thermodynamic Stability (Figure 7, Tables S3.9 and S6.1)

The bio-hybrid FTIR peaks intensities decreased by 0–26.9%, and the peak at 716 cm^−1^ did not change at all. T_g_ remained unchanged at 54.6 °C. Melting remained monomodal over the same temperature range as the initial bio-hybrid, with the same maximum and melting enthalpy. Crystallization occurred over the same range, and crystallinity was practically constant, as was crystallization enthalpy. HDT did not change, and density varied only at the second decimal place. SEM morphology remained inhomogeneous, crystallinity remained at 19% as well, and HDT was almost the same. Overall, the bio-hybrid seems not to have undergone morpho-structural changes and is most probably in a thermodynamic equilibrium state, meaning that it did not physically age over 2.2 years. 

### 3.4. Thermal Stability of the PLA Matrix (Figure S1.1, Tables S1.2 and S1.3)

Initial neat PLA has a Tg of 60.9 °C, cold crystallization with a maximum at 127 °C, and monomodal melting with a maximum at 153.2 °C. After 2.2 years, Tg increased to 66 °C (5.1 °C higher), the cold crystallization maximum shifted to 113°C (14 °C lower), and crystallization enthalpy increased by 29.9 J/g. Melting becomes bimodal, between 148 °C and 188 °C, with two maxima at 156 °C and 169 °C and a melting enthalpy of 32.33 J/g. After 2.2 years, neat PLA has polymorphic crystallinity, with large crystals coexisting with small ones, which need an additional melting enthalpy of 1.98 J/g. These crystals practically appear over the entire temperature range from 200 °C to 66 °C. Therefore, several small crystallization maxima are recorded on the cooling thermogram of PLA at 193 °C, 168 °C, 156 °C, 134 °C, 123 °C, 100 °C, 88 °C, and 66 °C. If neat PLA had a crystallinity of 0.08%, after 2.2 years, crystallinity increased to 2.7%, which means an increase of 2.62%. The results prove that even the PLA matrix changed over 2.2 years due to its tendency to reach thermodynamic equilibrium over time.

### 3.5. Scaling Up

Scale-up was performed with good results for the formulation with the nucleating agent.

## 4. Discussion

Polymer miscibility represents the thermodynamic ability of two or more polymers to form, at the molecular level, a single homogeneous phase with properties characteristic of a single material (e.g., a single glass transition [80]). Miscibility is governed by the change in free energy of blending, which is negative as a general rule [81], and is determined by blending thermodynamics, including the entropy of blending and enthalpic interactions between polymers [82,83]. On the other hand, compatibilization represents the better possible dispersion of phases in the polymeric matrix, with the help of compatibilizers acting as bridges between the dispersed phases and the matrix, which creates interfacial adhesion and thus improved functional properties [84]. From a practical point of view, it is much easier to lower interfacial tension using compatibilizers than to achieve thermodynamic miscibility at the molecular level [85].

The physical aging of the studied bio-hybrids depends fundamentally on the miscibility of the PLA matrix with the used components. If an amorphous system is miscible, physical aging can cause crystallization of another component, which diminishes amorphous content and hardening time of the bio-hybrid. In immiscible blends, a possible mechanism is inhibition of macromolecular rearrangement and delayed physical aging due to dispersed domains within the PLA matrix [86,87,88]. If the system is semi-crystalline, then the characteristics of physical aging depend on each individual situation

The observed cold crystallization in the PLA thermogram means that it was rapidly frozen without sufficient crystallization time, resulting in a glassy, amorphous, non-crystalline, disordered state. Upon heating to temperatures below the melting temperature, macromolecules rearrange into a more ordered state, representing a transition from an energetically charged amorphous state to a less energetically charged amorphous state those, namely the crystalline state [89]. The existence of cold crystallization therefore indicates a material with high susceptibility to physical aging. Over 2.2 years, the PLA matrix physically aged substantially: Tg increased by 5.1 °C, crystallinity increased to 2.7%, crystallization needed higher enthalpy of 33 J/g, and melting (still bimodal) shifted by 40 °C towards higher temperatures, which means the formation of larger crystals. Also, on the crystallization thermogram, a multitude of crystallization peaks exist, each corresponding to a different crystal size, which is another sign of physical aging during those 2.2 years. It should not be forgotten that the maintenance of cold crystallization over those 2.2 years indicates the availability of PLA for further physical aging.

The bio-hybrid with 16% PCL (RT108) has bimodal melting with two maxima, the first close to PCL melting and the second close to PLA melting. After 2.2 years, this composition shows clear signs of physical aging, as proven by increases in Tg, crystallinity, and melting and crystallization enthalpies. This behavior proves a non-equilibrium thermodynamic state that changes over time through macromolecular relaxation in an attempt to reach a less energetic state. It must not be forgotten that the morphology is predominantly amorphous, as crystallinity after 2.2 years is only 2.8% Initial FTIR analysis and after 2.2 years demonstrates some degree of interaction among the three components. However, considering all the results as a whole, including multiphase, polymorphic, and strongly inhomogeneous morphologies, it appears that melt compounding led to a PCL arrangement between PLA and talc that did not produce miscibility, possible only some degree of compatibilization. Given the presence of cold crystallization in neat PLA after 2.2 years, this bio-hybrid may continue to physically age. Despite its high HDT value and adequate mechanical behavior at 2.2 years, this bio-hybrid does not meet the objective for 3D-printed medium-life items.

The bio-hybrid containing 3.5% PCL (RT 93) has a glass transition temperature at 54 °C, cold crystallization with a maximum at 58 °C, broad bimodal melting between 162 °C and 128 °C, and crystallization occurring between 127 °C and 101 °C. This bio-hybrid has a Tg lower by 6 °C than that of the PLA matrix, and cold crystallization is preserved, which means that this composition still has availability for physical aging. This composition is not miscible and is in a state where interfacial tension is reduced by PCL, but not canceled. After 2.2 years, glass transition is nearly unchanged, melting range moves by 34 °C to lower temperatures, and crystallinity decreases by 6%, as do melting and crystallization enthalpies. These results attest that during the 2.2 years, the bio-hybrid has undergone an aging process due to crystal defects, not to the rearrangement of the amorphous phase. As described above, crystal defects can generate secondary crystallization, forming smaller crystals with variable sizes and in smaller quantities. Even though this formulation has a high HDT of practical interest and proper mechanical behavior after 2.2 years, it does not meet the objective for 3D-printed medium-life items.

A completely different behavior was achieved in the case of the bio-hybrid with 3.5% PCL and 3% nucleating agent (RT 103). All FTIR peaks of this bio-hybrid seem to result from interactions among the components. This composition has a single glass transition, no cold crystallization, a single monomodal melting, and a single crystallization event, with crystallinity around 20%. The small shoulder on the melting endotherm is easily correctable by formulation adjustment. The existence of a single melting event in a system containing at least two components that are almost immiscible under certain conditions (PLA and PCL) [90,91,92] shows that a thin PCL arrangement at the PLA and talc interface was achieved, favoring interfacial interactions. This composition has a high compatibility and even some degree of miscibility, which ensures thermodynamic stability over 2.2 years. Although the morphology of this bio-hybrid is still multiphase, it is stable over time. It is possible that crystallization, due to the nucleation of PLA macromolecules, on one hand, around the talc particles and, on the other hand, around the nucleating agent, has diminished the energy surplus of the amorphous phase through the formation of new crystals and thus ensured a relatively constant time behavior. This bio-hybrid is therefore compatible with 3D-printed medium-life applications.

Many solutions exist to improve the long-term behavior of these bio-hybrids. Data on physical aging and thermodynamic stability help support lifetime prediction of the bio-hybrids based on molecular simulation models and advanced statistical methods, with the help of artificial intelligence.

## 5. Conclusions

1. The aim of the article was to identify the dependence of the physical aging of PLA-talc-PCL bio-hybrids, achieved by melt compounding and designed for 3D-printed items with medium life, on the degree of miscibility, and to identify a formulation with a heat deflection temperature (HDT) of practical interest that is thermodynamically stable for at least two years. Melt compounding and 3D printing by the molten filament method require polymer blends processable in narrow temperature ranges, easily controllable, and with stable functional properties over time.
2. PLA was melt-compounded with PCL and talc in two variants, and with PCL, talc, and a nucleating agent in another. The bio-hybrids thus obtained were characterized morpho-structurally (FTIR, thermal analysis, XRD, SEM) and functionally (HDT, density, hardness, qualitatively estimated mechanical properties), initially and after 2.2 years. The preservation of properties over time was analyzed by examining physical aging, defined as all transformations generated by the relaxation of macromolecules in the amorphous phase and/or by crystal defects (interruption points of the ideal crystalline structure). Physical aging changes morpho-structural and functional properties and occurs as a transition over time from an energetic, thermodynamically unstable state to a less energetic, thermodynamically stable state. Because of physical aging, polymers ‘applications as structural components in the automotive field, e.g., or in electronics are not possible.
3. By melt compounding of PLA with PCL and talc, partially miscible or very poorly miscible bio-hybrids with multiphase, polymorphic morphology were achieved, which during 2.2 years of testing proved to be highly thermodynamically unstable. The physical aging of the bio-hybrid with 16% PCL was due to relaxation of macromolecules in the amorphous areas (initial crystallinity 5% and 6.8% after 2.2 years). whereas for the bio-hybrid with 3.5% PCL, it was due to crystal defects (initial crystallinity 29.7% and 23.4% after 2.2 years). In both cases, physical aging has a second cause, namely the thermodynamic instability of PLA. The thermodynamic instability of these two bio-hybrids makes them incompatible with 3D-printed items intended for medium-lifetime applications.
4. The use of a nucleating agent in the PLA, PCL, and talc formulation led to a bio-hybrid with a single monomodal melting peak in a narrow temperature range, which means good miscibility at the molecular level, thermodynamic stability over 2.2 years, and even the cancelation of PLA-induced instability. In this formulation, the positive compatibility effect of PCL, which ensured good miscibility at the interface, was added with the nucleating role of talc, in conjunction with that of the sulfonate compound (nucleating agent), for additional crystallinity control. In this way, it was possible to eliminate the energy surplus contained in the amorphous phase and to transform defective crystals into homogeneous crystals of small size. The initial crystallinity of this bio-hybrid was 19% initially and remained 19% after 2.2 years. This morphology ensured constant bio-hybrid behavior over time.
5. The physical aging of the bio-hybrids depended on the formulation through the PCL amount, the talc particle size distribution, the presence or absence of the nucleating agent in the formulation, and also on the availability of PLA macromolecules to relax over time. It is possible that, in addition to the nucleation effect of small talc particles, larger particles exerted a reinforcing influence, which has not yet been investigated. The morpho-structural and functional characterization of the selected formulation will be further investigated and possibly corrected to move to the next level of scaling up.
6. Being reliable, even if complex and time-consuming to estimate, thermodynamic stability data help predict the lifetime of compounds using molecular simulation models and advanced statistical methods. Artificial intelligence helps to model, predict, and understand the complex mechanisms involved in physical aging specific to polymers existing in a thermodynamic equilibrium of minimum energy charge.

## Figures and Tables

**Table 1:** Thermal behavior of the RT 93 bio-hybrid (glass transition, cold crystallization, melting).

Selected, Three-Component Bio-Hybrid with 3.5% PCL (RT93)																
Analysis Date	DSC Runs		Glass Trans., °C		Cold Crystallization (exo)						Melting (endo)					
			Tg, °C	ΔTg_2.2-i_, °C	Tcc, °C	ΔTcc_2.2-i_, °C	ΔHcc, J·g^−1^	ΔHcc_2.2-i_, J·g^−1^	R, °C	R_2.2-i_, °C	Tm, °C	ΔTm_2.2-i_, °C	ΔHm, J·g^−1^	ΔHm_2.2-i_, J·g^−1^	R, °C	R_2.2-i_, °C
Initial	Heating 2	Melt. 1 (M1.1/ Sh/M1.2)	54.6	-	58.8	-	0.5	-	39–64	25	158.5/-/169.7	-	28.1	-	128–179	51
After 2.2 years	Heating 2	Melt. 1 (M1.1/ Sh/M1.2)	54.4	0.2 ~	58.6	0.2 ~	0.5	0	38–63	25 ~	149.4/-/155	8.6↓/-/14.7↓	22.3	5.8↓	128–162	←34

~—approx. equal; ←—displacement towards left; ↓—decrease; Δ—variation; R—range; M—maximum; Sh—shoulder.

**Table 2:** Thermal behavior of the bio-hybrid with 3.5% PCL (RT 93) (crystallization).

Selected, Three-Component Bio-Hybrid with 3.5% PCL (RT93)									
Analysis Date	DSC Runs	Crystallization (exo)							
		T_c_, °C	ΔTc_2.2-i_, °C	ΔHc, J·g^−1^	ΔHc_2.2-i_, J·g^−1^	R, °C	R_2.2-i_., °C	C, %	ΔC_2.2-i_, %
Initial	Cooling	116.7	-	26.6	-	127–101	26	29.7	-
After 2.2 years	Cooling	108	8.7↓	21.4	5.2↓	127–98	←29	23.4	6.3↓

←—displacement towards left; ↓—decrease; Δ—variation; C—crystallinity; R—range.

**Table 3:** Thermal behavior of the bio-hybrid with 16% PCL (RT 108) (glass transition, cold crystallization, melting).

Selected, Three-Component Bio-Hybrid with 16% PCL (RT108)																		
Analysis Date	DSC Runs		Glass Trans., °C		Crystallization (exo)								Melting (endo)					
			T_g_, °C	ΔTg_2.2-i_, °C	T_c_, °C	ΔTc_2.2-i_, °C	ΔHc, J·g^−1^	ΔHc_2.2-i_, J·g^−1^	R, °C	R_2.2-i_., °C	C, %	ΔC, %	T_m_, °C	ΔTm_2.2-i_, °C	ΔHm, J·g^−1^	ΔHm_2.2-i_, J·g^−1^	R, °C	R_2.2-i_., °C
Initial	Cooling	Crystl. 1	-	-	107.5	-	3.2	-	115–100	15	-	-	-	-	-	-	-	-
		Crystl. 2	-	-	40.8	-	0.7	-	46–41	5	-	-	-	-	-	-	-	-
	Heating 2	Melt. 1	-	-	-	-	-	-	-	-	1.1	-	55.9	-	1	-	47–61	14
		Melt. 2 (M_2.1_/ Sh/M_2.2_)	-	-	-	-	-	-	-	-	3.9	-	149.9/ 155.8/-	-	3.6	-	127–162	35
After 2.2 years	Cooling	Crystl. 1	-	-	106	1.5↓	4.1	0.9 ~	114–98	←16	-	-	-	-	-	-	-	-
		Crystl. 2	-	-	41.6	0.8 ~	1.5	0.8 ~	46–34	←12	-	-	-	-	-	-	-	-
		Crystl. 3	-	-	28.9	-	0.2	0.2	31–27	4	-	-	-	-	-	-	-	-
	Heating 2	Melt. 1	-	-	-	-	-	-	-	-	2	0.9~	55.7	0.2 ~	1.9	0.9 ~	44–63	19→
		Melt. 2	-	-	-	-	-	-	-	-	-		-	-	-	-	-	-
		Melt. 3 (M_3.1_/ Sh/M_3.2_)	-	-	-	-	-	-	-	-	4.8	0.9~	148.2/ -/155.5	1.7↓/-/-	4.5	0.9 ~	127–162	35 ~

~—approx. equal; ←—displacement towards left; →—displacement towards right; ↓—decrease; Δ—variation; C—crystallinity; R—range; M—maximum; Sh—shoulder.

**Table 4:** Thermal behavior of the RT 103 bio-hybrid (glass transition, cold crystallization, melting).

Selected, Four-Component Bio-Hybrid with Nucleating Agent (RT103)																	
Analysis Date	DSC Runs	Glass Trans., °C		Crystallization (exo)								Melting (endo)					
		T_g_, °C	ΔTg_2.2-i_, °C	T_c_, °C	ΔTc_2.2-i_, °C	ΔHc, J·g^−1^	ΔHc_2.2-i_, J·g^−1^	R, °C	R_2.2-i_, °C	C, %	ΔC, %	T_m_, °C	ΔTm_2.2-i_, °C	ΔHm, J·g^−1^	ΔHm_2.2-i_, J·g^−1^	R, °C	R_2.2-i_., °C
Initial	Cooling	-	-	108.5	-	15.8	-	116–101	-	-	-	-	-	-	-	-	-
	Heating 2	54.6	-	-	-	-	-	-	-	19.3	-	149.8	-	17.9	-	130–159	-
After 2.2 years	Cooling	-	-	107.9	0.6 ~	15.6	0.2 ~	116–101	~	-	-	-	-	-	-	-	-
	Heating 2	54.6	0	-	-	-	-	-	-	19.1	0.2 ~	149	0.8 ~	17.7	0.2 ~	130–159	~

~—approx. equal; Δ—variation; C—crystallinity; R—Range.

## Data Availability

The original contributions presented in this study are included in the article/Supplementary Material. Further inquiries can be directed to the corresponding authors.

## Supplementary Materials

The following supporting information can be downloaded at: https://www.mdpi.com/article/10.3390/polym18010061/s1, Figure S1.1: Thermograms (glass transition (**a**), melting (**b**), heating (**c**), cooling (**d**)) of neat PLA; Figure S1.2: XRD diffractogram of neat PLA; Figure S1.3: SEM morphology of neat PLA; Figure S1.4: XRD diffractogram of talc; Figure S1.5: SEM morphology of talc; Figure S1.6: XRD diffractogram of nucleating agent; Figure S1.7: XRD diffractogram of PCL; Figure S1.8: Thermograms (melting (a), crystallization (b)) of PCL; Table S1.1: FTIR absorptions of neat PLA; Table S1.2: Thermal behavior of neat PLA (glass transition, cold crystallization, melting); Table S1.3: Thermal behavior of neat PLA (crystallization); Table S1.4: XRD data and crystallinity of neat PLA; Table S1.5: FTIR absorptions of talc; Table S1.6: XRD data and crystallinity of talc; Table S1.7: FTIR absorptions of nucleating agent; Table S1.8: XRD data of nucleating agent; Table S1.9: FTIR absorptions of PCL; Table S1.10: XRD data and crystallinity of PCL; Table S1.11: Thermal behavior of PCL (glass transition, cristalization, melting); Table S2.1: Formulations and durability of the studied bio-hybrids; Figure S3.1: FTIR spectra of bio-hybrids with different PLA-Talc-PCL formulations (a–e): low; (f–k): high; Table S3.1: FTIR changes of bio-hybrid with 3.5% PCL (RT 93); Table S3.2: FTIR absorptions of bio - hybrid with 3.5% PCL (RT 93); Table S3.3: FTIR changes of bio-hybrid with 3.5% PCL (RT 93) after 2.2 years; Table S3.4: FTIR modifications of bio–hybrid with 16% PCL (RT 108); Table S3.5: FTIR absorptions of bio-hybrid with 16% PCL (RT 108); Table S3.6: FTIR changes of bio - hybrid with 16% PCL (RT 108) after 2.2 years; Table S3.7: FTIR modifications of bio-hybrid with nucleating agent (RT 103); Table S3.8: FTIR absorptions of bio-hybrid with nucleating agent (RT 103); Table S3.9: FTIR changes of bio-hybrid with nucleating agent (RT 103) after 2.2 years; Table S4.1: XRD data and crystallinity of bio-hybrids (with 3.5% PCL, 16% PCL and with nucleating agent) initially and after 2.2 years; Figure S5.1: SEM morphology of bio-hybrid with 3.5% PCL (RT 93) after 2.2 years; Figure S5.2: SEM morphology of bio-hybrid with 16% PCL (RT 108) initially and after 2.2 years; Figure S5.3: SEM morphology of bio-hybrid with nucleating agent (RT 103) initial and after 2.2 years; Table S6.1. Physical properties of components and bio-hybrids (initial and after 2.2 years); Table S7.1. Mechanical behavior after 2.2 years of bio – hybrids with 3.5% PCL, 16% PCL and with nucleating agent. The following supporting information S1, S2, S3, S4, S5, S6, S7.

## References

[1] Ebrahimi F., Dana H.R. (2021). Poly lactic acid (PLA) polymers: From properties to biomedical applications. Int. J. Polym. Mater..

[2] Bikiaris N., Koumentakou I., Samiotaki C., Meimaroglou D., Varytimidou D., Karatza A., Kalantzis Z., Roussou M., Bikiaris R., Papageorgiou G. (2023). Recent advances in the investigation of poly (lactic acid) (PLA) nanocomposites: Incorporation of various nanofillers and their properties and applications. Polymers.

[3] Dana H.R., Ebrahimi F. (2022). Synthesis, properties, and applications of polylactic acid-based polymers. Polym. Eng. Sci..

[4] Mou L., Li J., Lu Y., Li G., Li J. (2025). Polylactic acid: A future universal biobased polymer with multifunctional performance—From monomer synthesis, and processing to applications: A review. J. Hazard. Mater. Adv..

[5] Ranakoti L., Gangil B., Mishra S.K., Singh T., Sharma S., Ilyas R., El-Khatib S. (2022). Critical review on polylactic acid: Properties, structure, processing, biocomposites, and nanocomposites, materials. Materials.

[6] Gzyra-Jagieła K., Sulak K., Draczyński Z., Podzimek S., Gałecki S., Jagodzińska S., Borkowski D. (2021). Modification of poly (lactic acid) by the plasticization for application in the packaging industry. Polymers.

[7] Simmons H., Tiwary P., Colwell J.E., Kontopoulou M. (2019). Improvements in the crystallinity and mechanical properties of PLA by nucleation and annealing. Polym. Degrad. Stab..

[8] Sun H., Luo W., Weng Y., Zhang C. (2025). Advances in poly (lactic acid) chain extenders: Mechanisms, performance, and sustainability. J. Vinyl Addit. Technol..

[9] Zhai S., Liu Q., Zhao Y., Sun H., Yang B., Weng Y. (2021). A review: Research progress in modification of poly (lactic acid) by lignin and cellulose. Polymers.

[10] Andrzejewski J., Das S., Lipik V., Mohanty A.K., Misra M., You X., Tan L.P., Chang B.P. (2024). The development of poly (lactic acid) (PLA)-based blends and modification strategies: Methods of improving key properties towards technical applications—Review. Materials.

[11] Giammaria V., Capretti M., Del Bianco G., Boria S., Santulli C. (2024). Application of poly(lactic acid) composites in the automotive sector: A critical review. Polymers.

[12] Bouzouita A. (2016). Elaboration of Polylactide-Based Materials for Automotive Application: Study of Structure-Process-Properties Interactions. Ph.D. Thesis.

[13] Akampumuza O., Wambua P.M., Ahmed A., Li W., Qin X. (2016). Review of the applications of biocomposites in the automotive industry. Polym. Compos..

[14] Glaskova-Kuzmina T., Starkova O., Gaidukovs S., Platnieks O., Gaidukova G. (2021). Durability of biodegradable polymer nanocomposites. Polymers.

[15] Muguda S., Lucas G., Hughes P., Augarde C., Perlot C., Bruno A., Gallipoli D. (2020). Durability and hygroscopic behaviour of biopolymer stabilised earthen construction materials. CBM.

[16] Homam S.M. Durability of fibre reinforced polymers used in concrete structures. Proceedings of the 3rd International Conference on Advanced Composite Materials in Bridges and Structures.

[17] Ehrenstein G., Pongratz S. (2013). Resistance and stability of polymers. Resistance and Stability of Polymers.

[18] Florjančič U., Emri I. (2008). Tailoring functionality and durability of polymeric products by modifying processing conditions. Stroj. Vestn..

[19] White J.R. (2006). Polymer ageing: Physics, chemistry or engineering? Time to reflect. Comptes Rendus Chim..

[20] Hutchinson J.M. (1995). Physical aging of polymers. Prog. Polym. Sci..

[21] Nunes S.G., Joffe R., Emami N., Fernberg P., Saseendran S., Esposito A., Amico S., Varna J. (2022). Physical aging effect on viscoelastic behavior of polymers. Compos. Part C.

[22] Minguez R., Barrenetxea L., Solaberrieta E., Lizundia E. (2018). A simple approach to understand the physical aging in polymers. Eur. J. Phys..

[23] Godard M.E., Saiter J.M., Burel F., Bunel C., Cortes P., Montserrat S., Hutchinson J.M. (1996). Physical aging in polymers: Comparison of two ways of determining Narayanaswamy’s parameter. Polym. Eng. Sci..

[24] Struik L.C.E. (1989). Mechanical behaviour and physical ageing of semi-crystalline polymers: 4. Polymer.

[25] Cangialosi D. (2018). Physical aging of polymers. Encycl. Polym. Sci. Technol..

[26] Skotnicki M., Drogoń A., Lulek J., Pyda M. (2023). Physical ageing of amorphous poly (lactic acid)-indapamide system studied by differential scanning calorimetry. Pharmaceutics.

[27] Muller P., Imre B., Bere J., Moczo J., Pukanszky B. (2015). Physical ageing and molecular mobility in PLA blends and composites. J. Therm. Anal. Calorim..

[28] Cui L., Imre B., Tátraaljai D., Pukánszky B. (2020). Physical ageing of poly (lactic acid): Factors and consequences for practice. Polymer.

[29] Orellana-Barrasa J., Ferrández-Montero A., Ferrari B., Ygnacio Pastor J. (2022). Natural ageing of PLA filaments, Can It Be Frozen?. Polymers.

[30] Zhong X., Qiang L., Cheng J., Sun Z., Hu H., Liu H., Zhang R. (2025). Aging or degradation? Transformation mechanisms of microplastics in soil environments. Appl. Soil Ecol..

[31] Zhao Y., Wan S., Xu M., Wu G., Wang D., Yi C., Cui L. (2025). Effect of aging on the properties of microplastics and their adsorption behavior of norfloxacin. J. Water Process. Eng..

[32] Burgos N., Martino V.P., Jiménez A. (2013). Characterization and ageing study of poly (lactic acid) films plasticized with oligomeric lactic acid. Polym. Degrad. Stab..

[33] Martino V.P., Ruseckaite R.A., Jiménez A. (2009). Ageing of poly (lactic acid) films plasticized with commercial polyadipates. Polym. Int..

[34] Ljunberg N., Wesslén B. (2003). Tributyl citrate oligomers as plasticizers for poly (lactic acid): Thermo-mechanical film properties and aging. Polymers.

[35] Dimonie D., Radovici C., Zaharia C., Vasilievici G., Stoleriu A. (2006). Thermal behaviour of biodegradable nanocomposites on the basis of polyvinylic alcohol and starch. Mater. Plast..

[36] Lagerlof P. (2018). Crystal dislocations: Their impact on physical properties of crystals. Crystals.

[37] Gahleitner M., Fiebig J., Wolfschwenger J. (2022). Post-crystallization and physical ageing of polypropylene: Material and processing effects. J. Macromol. Sci. Part B.

[38] Hay J.N. (1995). The physical ageing of amorphous and crystalline polymers. Pure Appl. Chem..

[39] Matsui M., Masur R., Wada Y. (1971). Defects caused by chain ends in polymer crystals and interpretation of plastic deformation and r-relaxation in terms of them. Polym. J..

[40] Cheng X., Wang S., Zhang X., Iqbal S., Yang Z., Xiang X. (2024). Accelerated aging behavior of degradable and non-degradable microplastics via advanced oxidation and their adsorption characteristics towards tetracycline. Ecotoxicol. Environ. Saf..

[41] Vashchuk A., Missaoui T., Delpouve N., Dargent E. (2023). Accelerated aging of 18 years rejuvenated polylactide bottles by fast scanning calorimetry. Appl. Res..

[42] De Santis F. (2015). Melt compounding of poly (lactic Acid) and talc: Assessment of material behavior during processing and resulting crystallization. J. Polym. Res..

[43] Zhang J. (2024). Preparation of PLA/PBAT blends with high performance via the synergistic effect of high mold temperature and strong shear field. Polymers.

[44] Lee C., Pang M.M., Koay S.C. (2020). Talc filled polylactic-acid biobased polymer composites: Tensile, thermal and morphological properties. SN Appl. Sci..

[45] Shakoor A. (2014). Talc as a nucleating agent and reinforcing filler in poly (lactic acid) composites. Polym. Eng Sci..

[46] Matumba K.I., Mokhena T.C., Ojijo V., Sadiku E., Ray S. (2024). Morphological characteristics, properties, and applications of polylactide/poly(ε-caprolactone) blends and their composites—A review. Macromol. Mater. Eng..

[47] Negaresh M., Javadi A., Garmabi H. (2024). Poly (lactic acid)/poly(ε-caprolactone) blends: The effect of nanocalcium carbonate and glycidyl methacrylate on interfacial characteristics. Front. Mater..

[48] Solechan S., Suprihanto A., Widyanto S.A., Triyono J., Fitriyana D.F., Siregar J., Cionita T. (2022). Investigating the effect of PCL concentrations on the characterization of PLC polymeric blends for biomaterial applications. Materials.

[49] Donate R., Paz R., Quintana A., Bordón P., Monzón M. (2023). Calcium carbonate coating of 3D-printed PLA scaffolds intended for biomedical applications. Polymers.

[50] Garcia D., Carbonell-Verdu A., Arrieta M.P., López-Martínez J., Samper M.D. (2020). Improvement of PLA film ductility by plasticization with epoxidized karanja oil. Polym. Degrad. Stab..

[51] Mastalygina E.E., Aleksanyan K.V. (2024). Recent approaches to the plasticization of poly (lactic acid) (PLA) (a review). Polymers.

[52] Liu L.J., Chen F.Y., Zhu S., Yan L., Zhao R.X., Chen P.Y., Wang B. (2025). Multi-functional polyvinyl chloride plastic additive based on epoxidized linseed oil-polycaprolactone: Preparation and application performance study. React. Funct. Polym..

[53] Bingzhe D., Jian G., Guangqiang X., Hongbin H., Rulin Y., Xuanhua G., Qinggang W. (2024). Trash into treasure: Chemical upcycling of poly(ε-caprolactone) waste plastic to plasticizer with excellent plasticizing performance and migration resistance. Sustain. Mater. Technol..

[54] Homaeigohar S., Boccaccini A.R. (2022). Nature-derived and synthetic additives to poly(ε-caprolactone) nanofibrous systems for biomedicine. Front. Chem..

[55] Ferri J.M., Fenollar O., Jorda-Vilaplana A., García Sanoguera D., Balart Gimeno R. (2016). Effect of miscibility on mechanical and thermal properties of poly (lactic acid)/polycaprolactone blends. Polym. Int..

[56] Wang Y., He D., Wang X., Cao W., Li Q., Shen C. (2013). Crystallization of poly (lactic acid) enhanced by phthalhydrazide as nucleation agent. Polym. Bull..

[57] Spinelli G., Kotsilkova R., Ivanov E., Petrova-Doycheva I., Menseidov D., Georgiev V., Di Maio R., Silvestre C. (2020). Effects of filament extrusion, 3D printing and hot-pressing on electrical and tensile properties of poly(lactic) acid composites filled with carbon nanotubes and graphene. Nanomaterials.

[58] (2019). Plastics—Methods for Determining the Density of Non-Cellular Plastics.

[59] (2018). Rubber, Vulcanized or Thermoplastic—Determination of Hardness.

[60] (2020). Plastics—Determination of Temperature of Deflection Under Load.

[61] Takeshita H., Shiomi T., Takenaka K., Arai F. (2013). Crystallization and higher-order structure of multicomponent polymeric systems. Polymers.

[62] Maleki F. (2021). Proliferation and osteogenic differentiation of mesenchymal stem cells on three-dimensional scaffolds made by thermal sintering method. Chem. Pap..

[63] Kemala T., Budianto E., Soegiyono B. (2010). Preparation and characterization of microspheres based on blend of poly (lactic acid) and poly(ε-caprolactone) with poly (vinyl alcohol) as emulsifier. Arab. J. Chem..

[64] Kawaguchi K. FTIR Spectroscopy of NO_3: Observation and Analysis of the 1127 cm^−1^ Band. Proceedings of the 65th International Symposium on Molecular Spectroscopy.

[65] Suárez Franco J.L., Vázquez-Vázquez F.C., Pozos-Guillen A., Montesinos J.J., Alvarez-Fregoso O., Alvarez-Perez M.A. (2018). Influence of diameter of fiber membrane scaffolds on the biocompatibility of hPDL mesenchymal stromal cells. Dent. Mater. J..

[66] Kondo Y., Goto T., Asano K., Nishida H., Cho S.H., Yin S., Sekino T. (2025). Photoactivation of surface peroxides on titanate nanotubes. Inorg. Chem..

[67] Bocchini S., Frache A. (2013). Comparative study of filler influence on polylactide photooxidation. Express Polym. Lett..

[68] Xiang L. (2018). Precise synthesis, properties, and structures of cyclic poly(ε-caprolactone) s. Polymers.

[69] Marwat Z., Baloch M. (2015). Solvent induced miscibility between polymers, and its influence on the morphology, and mechanical properties of their blends. Eur. Polym. J..

[70] He J., Liu J. (1999). Miscibility enhancement of modified polystyrene blends with a liquid crystalline polymer. Polymer.

[71] Icoz D.Z., Kokini J.L. (2007). Probing the boundaries of miscibility in model carbohydrates consisting of chemically derivatized dextrans using DSC and FTIR spectroscopy. Carbohydr. Polym..

[72] Dimonie D., Radu S., Doncea S., Pop F.S., Petre C., Dumitriu I., Fierascu R. (2011). The miscibility estimation of some nanocomposites based on starch. e-Polymers.

[73] Przybysz-Romatowska M., Haponiuk J., Formela K. (2020). *Poly(ε*-caprolactone)/poly (lactic acid) blends compatibilized by peroxide initiators: Comparison of two strategies. Polymers.

[74] Shojaei S. (2018). Disclosing the role of surface and bulk erosion on the viscoelastic behavior of biodegradable poly(ε-caprolactone)/poly (lactic acid)/hydroxyapatite nanocomposites. J. Appl. Polym. Sci..

[75] Braun B. (2006). Infrared spectroscopic determination of lactide concentration in polylactide: An improved methodology. Macromolecules.

[76] Shimadzu Corporation *Application News, Spectrophotometric Analysis*; No.A422; International Marketing Division: Mumbai, Maharashtra. https://www.shimadzu.com/an/sites/shimadzu.com.an/files/pim/pim_document_file/applications/application_note/11836/a422.pdf.

[77] Liang J., Yang B., Deng J. (2018). Polylactide-based chiral particles with enantio-differentiating release ability. Chem. Eng. J..

[78] Tazibt N., Kaci M., Dehouche N., Ragoubi M., Atanase L.I. (2023). Effect of filler content on the morphology and physical properties of poly (lactic acid)-hydroxyapatite composites. Materials.

[79] Marzbani P., Resalati H., Ghasemian A., Shakeri A. (2016). Surface modification of talc particles with phthalimide: Study of composite structure and consequences on physical, mechanical, and optical properties of deinked pulp. BioResources.

[80] Arrighi V., Cowie J.M., Fuhrmann S., Youssef A., Isayev A.I. (2010). Miscibility criterion in polymer blends and its determination. Encyclopedia of Polymer Blends.

[81] Olabisi O. (1981). Interpretations of polymer-polymer miscibility. J. Chem. Educ..

[82] Olabisi O., Robeson L.M., Shaw M.T. (1979). Polymer-Polymer Miscibility.

[83] Angeli C.A. (1995). Formation of glasses from liquids and biopolymers. Science.

[84] Utracki L.A. (2002). Compatibilization of polymer blends. Can. J. Chem. Eng..

[85] Fredrickson G.H., Xie S., Edmund S., Le M., Sun D. (2022). Ionic compatibilization of polymers. ACS Polym. Au.

[86] Liu Q., Zhang H., Zhu M., Dong Z., Wu C., Jiang J., Li X., Luo F., Gao Y., Deng B. (2013). Blends of polylactide/thermoplactic elastomer: Miscibility, physical aging and crystallization behaviors. Fibers Polym..

[87] Höglund A., Hakkarainen M., Edlund U., Albertsson A.-C. (2010). Surface modification changes the degradation process and degradation product pattern of polylactide. Langmuir.

[88] Sedničková M., Pekařová S., Kucharczyk P., Bočkaj J., Janigová I., Kleinová A., Jochec-Mošková D., Omaníková L., Perďochová D., Koutný M. (2018). Changes of physical properties of PLA-based blends during early stage of biodegradation in compost. Int. J. Biol. Macromol..

[89] Ishino K., Yasuy H. (2021). Cold crystallization and the molecular structure of imidazolium based ionic liquid crystals with a np-nitroazobenzene moiety. ACS Omega.

[90] Gao P., Woodward D., Johnson M., Gajkowski N., Kaminsky L. Effects of injection molding parameters and orotic acid on mechanical and thermal properties of poly lactic acid. Proceedings of the Conference Proceedings.

[91] Barletta M., Pizzi E., Puopolo M., Vesco S. (2017). Design and manufacture of degradable polymers: Biocomposites of micro-lamellar talc and poly(lactic acid). Mater. Chem. Phys..

[92] Dimonie D., Dragomir N., Stoica R. (2021). Attempts to diminish the drawbacks of polylactic acid designed for 3D/4D printing technology-fused deposition modeling. Mater. Plast..

