# Supplementary material for: The Influence of Miscibility of Some PLA-Based Bio-Hybrids Designed for 3D Printing and Medium-Life Applications on Their Physical Aging and Thermodynamic Stability
Source: Polymers (Basel). 2025 Dec 25;18(1):61. doi: 10.3390/polym18010061 (PMC12788091; doi:10.3390/polym18010061)
Supplement: Supplementary file 1 [file polymers-18-00061-s001.zip › Supplementary Material 1 (S1).pdf]

Supplementary Material 1_Neat PLA (S1_Neat PLA)

Table S1.1. FTIR absorptions of neat PLA

Component	Wavenumber, cm^{-1} / Absorbance, a.u.
Neat PLA 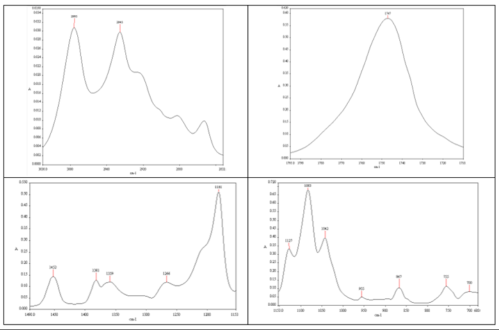	2995 cm^{-1} / 0.031; 2945 cm^{-1} / 0.030; 2928 cm^{-1} / 0.021; 2900 cm^{-1} / 0.012; 2880 cm^{-1} / 0.011; 2851 cm^{-1} / 0.010; 1747 cm^{-1} / 0.580; 1452 cm^{-1} / 0.140; 1381 cm^{-1} / 0.130; 1359 cm^{-1} / 0.120; 1310 cm^{-1} / 0.040; 1266 cm^{-1} / 0.120; 1210 cm^{-1} / 0.270; 1181 cm^{-1} / 0.510; 1127 cm^{-1} / 0.330; 1083 cm^{-1} / 0.680; 1042 cm^{-1} / 0.400; 955 cm^{-1} / 0.040; 867 cm^{-1} / 0.100; 755 cm^{-1} / 0.110; 700 cm^{-1} / 0.070;

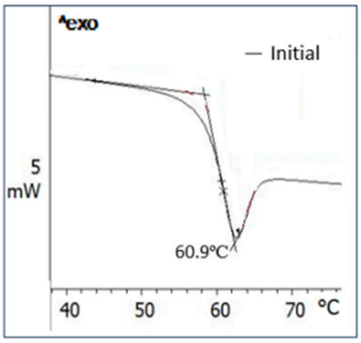

(a)

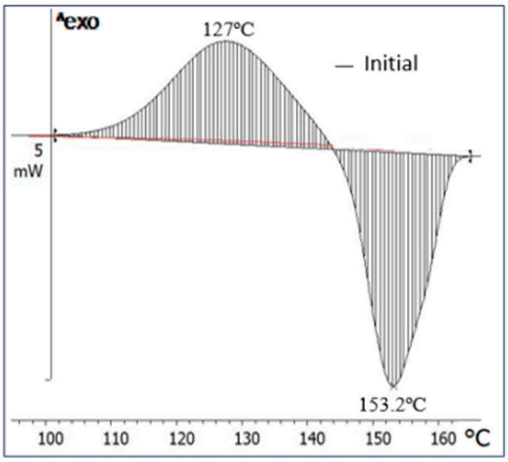

(b)

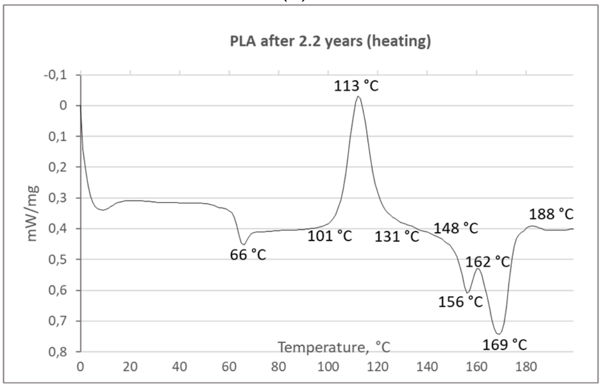

(c)

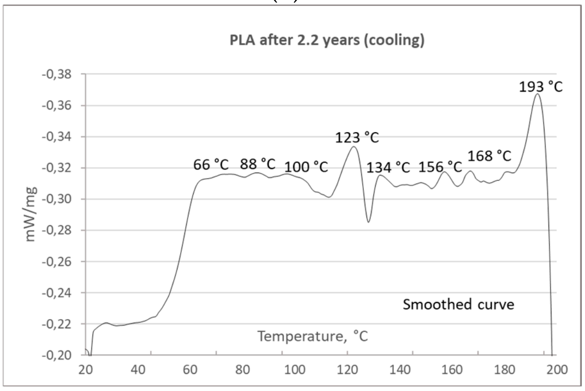

(d)

Figure S1.1. Thermograms (glass transition (**a**), melting (**b**), heating (**c**), cooling (**d**)) of neat PLA

Table S1.2. Thermal behavior of neat PLA (glass transition, cold crystallization, melting)

Neat PLA															
**Analysis date**	DSC runs	**Glass Trans., °C**		**Cold crystallization(exo)**						**Melting (endo)**					
		T_g, °C	$\Delta Tg_{2.2\text{-}i}$, °C	T_{cc}, °C	$\Delta T_{cc2.2\text{-}i}$, °C	ΔH_{cc}, J·g^{-1}	$\Delta Hcc_{2.2\text{-}i}$, J·g^{-1}	R,°C	$R_{2.2\text{-}i.}$, °C	T_m,°C	$\Delta Tm_{2.2\text{-}i}$, °C	ΔHm, J·g^{-1}	$\Delta Hm_{2.2\text{-}i}$, J·g^{-1}	R,°C	$R_{2.2\text{-}i.}$, °C
**Initial**	Heating 2	60.9	-	127	-	0.7	-	101-145	44	153.2	-	0.77	-	145-165	20
**After 2.2 years**	Heating 2	66↑	5.1	113	14↓	30.6	29.9↑	101-131	30←	156/169	2.8↑	33.1	32.33↑	148-188	40→

~ - aprox. equal; ← - displacement toward left; → - displacement towards right; ↑ - increase; ↓- decrease; Δ – variation; C- crystallinity; R-Range.

Table S1.3. Thermal behavior of neat PLA (crystallization)

Neat PLA									
**Analysis date**	**DSC runs**	**Crystallization(exo)**							
		T_c, °C	$\Delta Tc_{2.2\text{-}i}$, °C	ΔHc, J·g^{-1}	$\Delta Hc_{2.2\text{-}i}$, J·g^{-1}	R, °C	$R_{2.2\text{-}i.}$, °C	C, %	$\Delta C_{2.2\text{-}i}$, %
**Initial**	Cooling	-	-	-	-	-	-	0.08	-
**After 2.2 years***	Cooling	123	123	1.98	1.98	113-128	15	2.7	2.62↑

~ - aprox. equal; ← - displacement toward left; → - displacement towards right; ↑ - increase; ↓- decrease; Δ – variation; C- crystallinity; R-Range.

* Considering only the main crystallization, not all the small peaks from 200°C - 60°C range.

Table S1.4. XRD data and crystallinity of neat PLA

**Component**	**Diffractions (angle, 2θ° and intensity, counts/s)**	**Total peaks no.**	**Crystallinity %**
Neat PLA	20° - 8000 cps; 22° - 35000 cps; 24° - 8000 cps; 27° -7500 cps; 34° - 6000 cps;	5	0.08 initial
			2.7 after 2.2 years

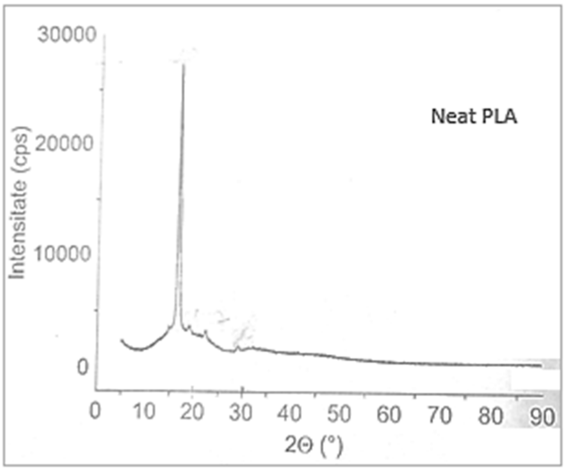

Figure S1.2. XRD diffractogram of neat PLA

Neat PLA

1000X **2000X** **4000X** **10000X**

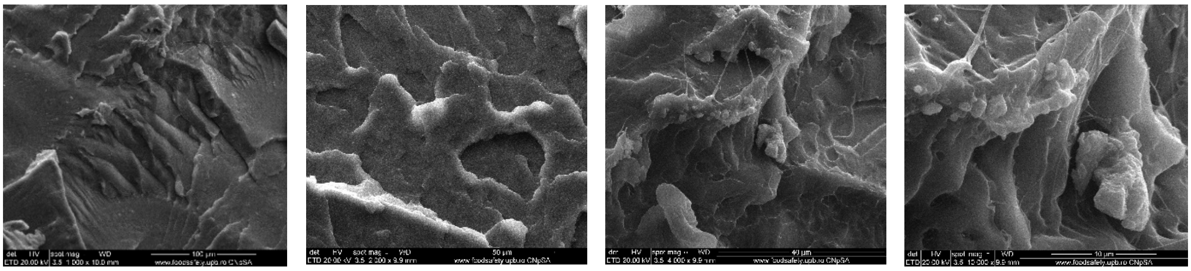

Figure S1.3. SEM morphology of neat PLA

Supplementary Material 1_Talc (S1_Talc)

Table S1.5. FTIR absorptions of talc

Component	Wavenumber, cm^{-1} / Absorbance, a.u.
Talc 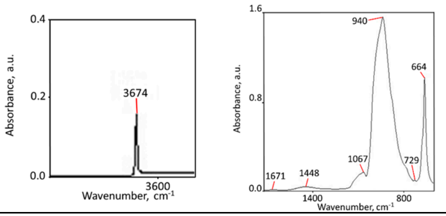	3674 cm^{-1} / 0.150; 1671 cm^{-1} / 0.010; 1448 cm^{-1} / 0.050; 1067 cm^{-1} / 0.150; 940 cm^{-1} / 1.550; 729 cm^{-1} / 0.100; 664 cm^{-1} / 1.000;

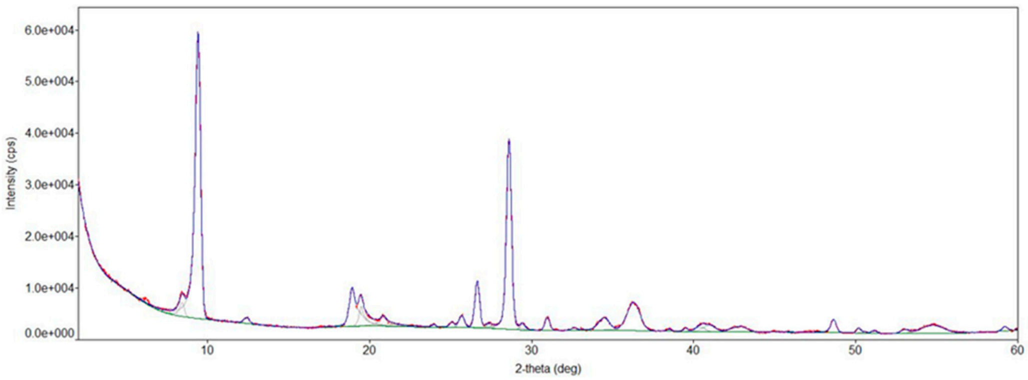

Figure S1.4. XRD diffractogram of talc

Table S1.6. XRD data and crystallinity of talc

Component	Diffractions (angle, 2θ° and intensity, counts/s)	Total peaks no.	Crystallinity %
Talc	8° - 9000 cps; 9° - 59000 cps; 12° - 3000 cps; 18° - 10000 cps; 19° – 9000 cps; 21° - 3000 cps; 25° - 5000 cps; 26° - 11000 cps; 28° - 39000 cps; 29° - 2500 cps; 31° - 5000 cps; 34° - 4000 cps; 36° - 8500 cps; 41° - 2500 cps; 43° - 2300 cps; 48° - 4000 cps; 55° - 2500 cps	17	Ideal 100%
			Commercial 10% - 95%

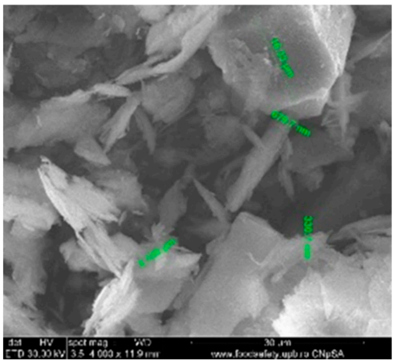

Figure S1.5. SEM morphology of talc

Supplementary Material 1_LAK 301 (S1_LAK 301)

Table S1.7. FTIR absorptions of nucleating agent

**Component**	**Wavenumber, cm^{-1} / Absorbance, a.u.**
LAK 301 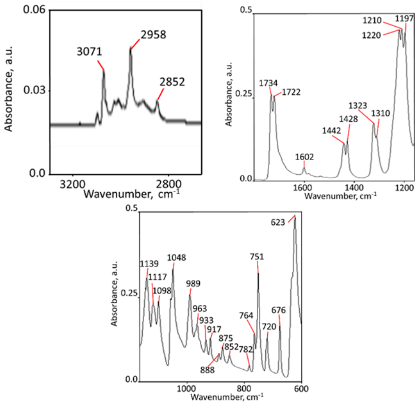	3070 cm^{-1} / 0.040; 2957 cm^{-1} / 0.046; 1734 cm^{-1} / 0.257; 1722 cm^{-1} / 0.255; 1601 cm^{-1} / 0.046; 1442 cm^{-1} / 0.110; 1428 cm^{-1} / 0.120; 1322 cm^{-1} / 0.177; 1219 cm^{-1} / 0.448; 1210 cm^{-1} / 0.450; 1196 cm^{-1} / 0.440; 1139 cm^{-1} / 0.310; 1117 cm^{-1} / 0.220; 1098 cm^{-1} / 0.240; 1048 cm^{-1} / 0.330; 988 cm^{-1} / 0.260; 963 cm^{-1} / 0.177; 932 cm^{-1}/ 0.140; 917 cm^{-1} / 0.140; 887 cm^{-1} / 0.090; 875 cm^{-1} / 0.100; 851 cm^{-1} / 0.085; 782 cm^{-1}/ 0.060; 764 cm^{-1} / 0.150; 751 cm^{-1} / 0.320; 720 cm^{-1} / 0.140; 676 cm^{-1} / 0.170; 623 cm^{-1} / 0.480.

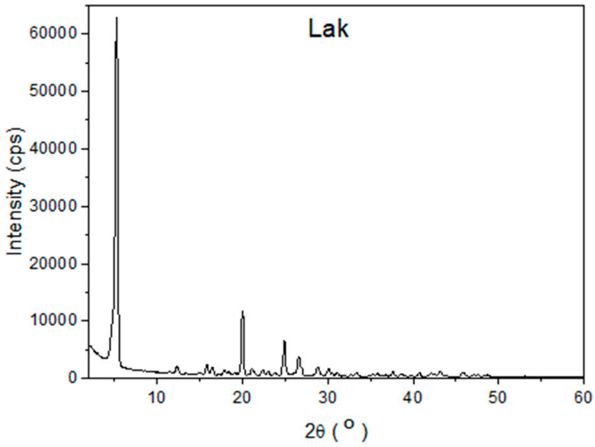

Figure S1.6. XRD diffractogram of nucleating agent

Table S1.8. XRD data of nucleating agent

**Component**	**Diffractions (angle, 2θ° and intensity, counts/s)**	**Total peaks no.**	**Crystallinity %**
LAK 301	5° - 66000 cps; 12° - 2000 cps; 16° -2500 cps; 20° - 12000 cps; 21 - 2000 cps; 22 - 1800 cps; 23 - 1750 cps; 24 - 1700 cps; 25 - 7000 cps; 27 - 4000 cps; 29 - 2000 cps; 30 - 2500 cps; 41 - 1750 cps; 43 - 1800 cps; 46 - 1800 cps	15	-

Supplementary Material 1_PCL (S1_PCL)

Table S1.9. FTIR absorptions of PCL

Component	Wavenumber, cm^{-1}/ Absorbance, a.u.
PCL600 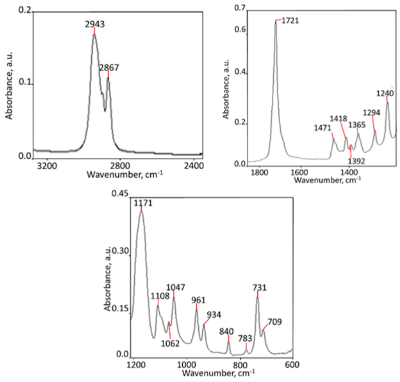	2943 cm^{-1} / 0.170; 2867 cm^{-1} / 0.120; 1721 cm^{-1} / 0.650; 1471 cm^{-1} / 0.120; 1418 cm^{-1} / 0.120; 1392 cm^{-1} / 0.090; 1365 cm^{-1} / 0.150; 1294 cm^{-1} / 0.150; 1240 cm^{-1} / 0.280; 1171 cm^{-1} / 0.420; 1108 cm^{-1} / 0.160; 1062 cm^{-1} / 0.120; 1047 cm^{-1} / 0.190; 961 cm^{-1} / 0.150; 934 cm^{-1} / 0.120; 840 cm^{-1} / 0.050; 783 cm^{-1} / 0.020; 731 cm^{-1} / 0.190; 709 cm^{-1} / 0.080;

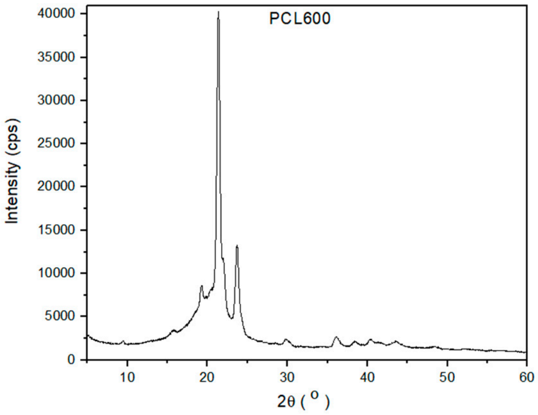

Figure S1.7. XRD diffractogram of PCL

Table S1.10. XRD data and crystallinity of PCL

Component	Diffractions (angle, 2θ° and intensity, counts/s)	Total peaks no.	Crystallinity %
PCL	19° - 8500 cps; 21° - 40000 cps; 24° - 13000 cps; 30° - 2000 cps; 36° - 3000 cps; 38° - 2500 cps; 41° - 2600 cps; 43° - 2600 cps	8	60

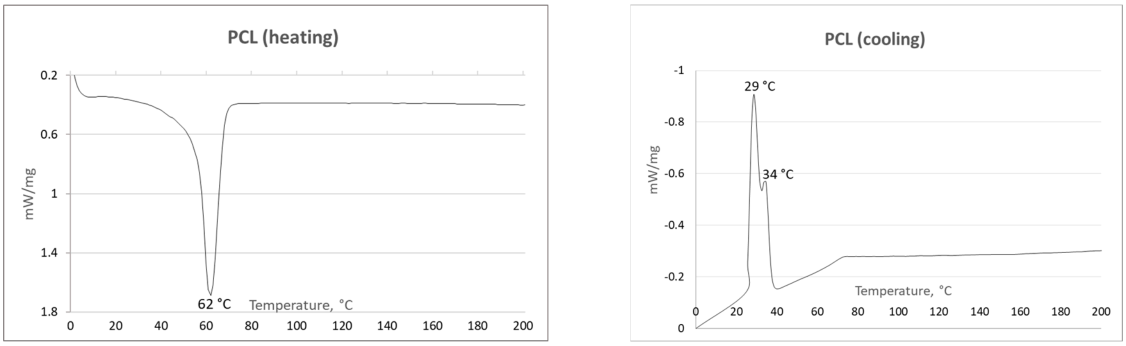

(a) (b)

Figure S1.8. Thermograms (melting (a), crystallization (b)) of PCL

Table S1.11. Thermal behavior of PCL (glass transition, cristalization, melting)

PCL								
**DSC runs**	**Glass Trans., °C**	**Crystallization(exo)**				**Melting (endo)**		
	Tg, °C	Tc,°C	ΔHc, J·g−1	R,°C	C, %	Tm,°C	ΔHm, J·g−1	R,°C
Cooling (M/Sh)	-	29/34	64.4	42-22	-	-	-	-
Heating 2	-	-	-	-	60	62	81	15-75

Δ – variation; C- crystallinity; R-range; M - maximum; Sh – shoulder.
