# Supplementary material for: The Influence of Miscibility of Some PLA-Based Bio-Hybrids Designed for 3D Printing and Medium-Life Applications on Their Physical Aging and Thermodynamic Stability
Source: Polymers (Basel). 2025 Dec 25;18(1):61. doi: 10.3390/polym18010061 (PMC12788091; doi:10.3390/polym18010061)
Supplement: Supplementary file 1 [file polymers-18-00061-s001.zip › Supplementary Material 2 (S2).pdf]

Supplementary Material 2 (S2)

Table S2.1. Formulations and durability of the studied bio-hybrids

Bio-hybrids code	Formulations		Durab., years
	[p]	%	
RT 88	15[p] talc / 1 [p] PCL	13% / 0.8%	2.2
RT 92	40[p] talc / 1[p] PCL	28% / 0.7%	2.2
RT 94	75[p] talc / 1 [p] PCL	42% / 0.6%	2.2
RT 89	15[p] talc / 5 p PCL	12.5% / 4%	4
RT 91	25[p] talc / 5[p] PCL	19% / 3.84%	4
RT 93	40[p] talc / 5[p] PCL	28% / 3.5%	2.2
RT 106	25[p] talc / 15 [p]PCL	18% / 11%	2.2
RT 107	25[P] talc / 25[P] PCL	17% / 17%	2.2
RT 108	25[p] talc / 30[p]PCL	17% / 21%	4
RT 103	75[p] talc / 5[p] PCL / 6[p] LAK 301	40% / 3% / 35%	4
