# Supplementary material for: The Influence of Miscibility of Some PLA-Based Bio-Hybrids Designed for 3D Printing and Medium-Life Applications on Their Physical Aging and Thermodynamic Stability
Source: Polymers (Basel). 2025 Dec 25;18(1):61. doi: 10.3390/polym18010061 (PMC12788091; doi:10.3390/polym18010061)
Supplement: Supplementary file 1 [file polymers-18-00061-s001.zip › Supplementary Material 3 (S3).pdf]

Supplementary material 3 (S3)

1. First selection (Bio-hybrids: PLA-Talc-PCL)

Legend:

- **- Black** - 15 [p] talc / 1 [p] PCL /100 [p] PLA;
- **- Pink** - 40 [p] talc / 1 [p] PCL / 100 [p] PLA;
- **- Green** - 75 [p] talc / 1[p] PCL /100 [p] PLA ;
- **- Red** - 15 [p] talc / 5 [p] PCL /100 [p] PLA;
- - **Blue** - 25 [p] talc / 5[p] PCL /100 [P] PLA;
- **- Turquoise** - 40 [p] talc / 5 [p] PCL /1000 [P] PLA.

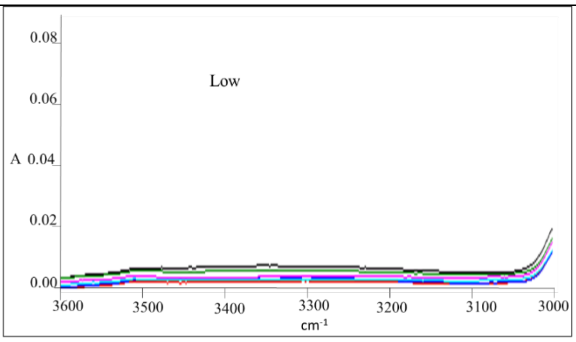

(a)

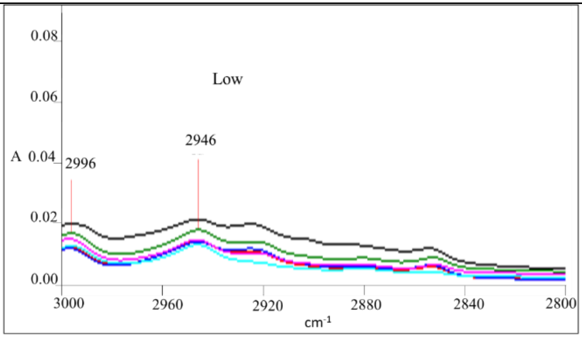

(b)

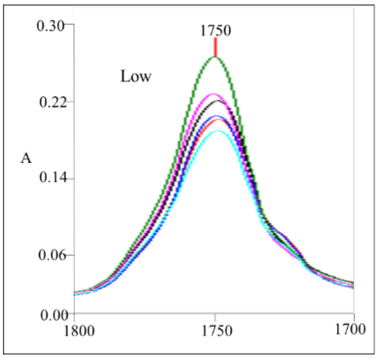

(c)

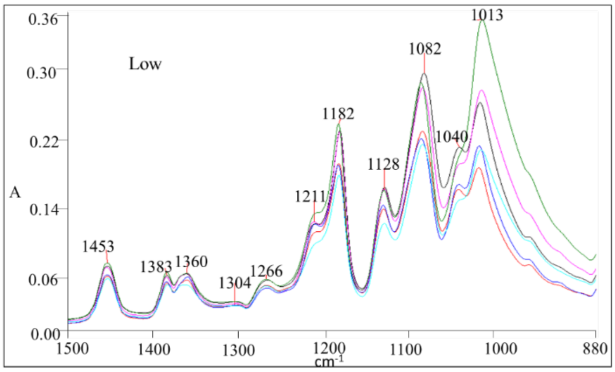

(d)

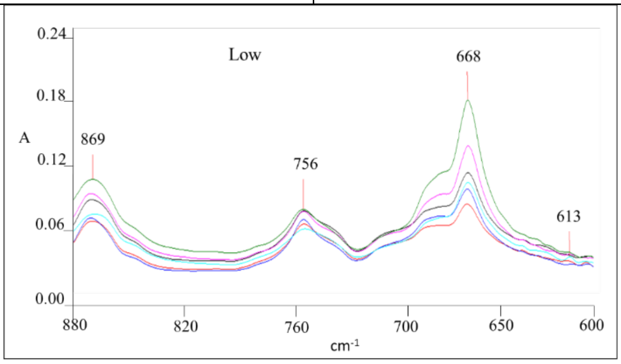

(e)

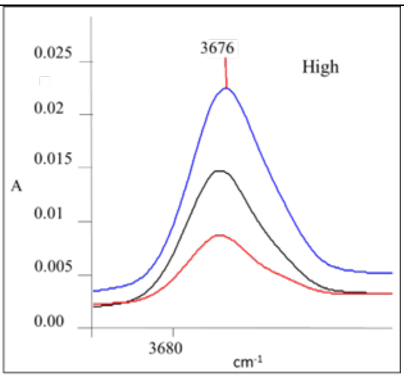

(f)

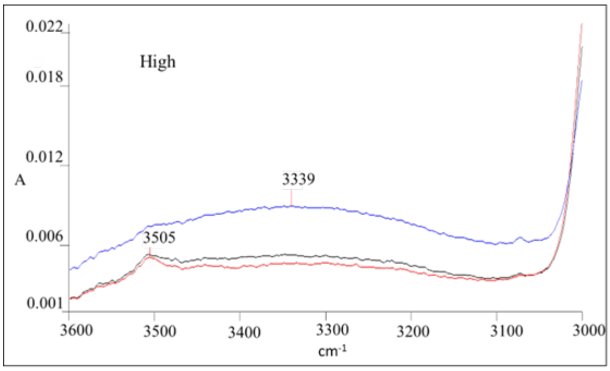

(g)

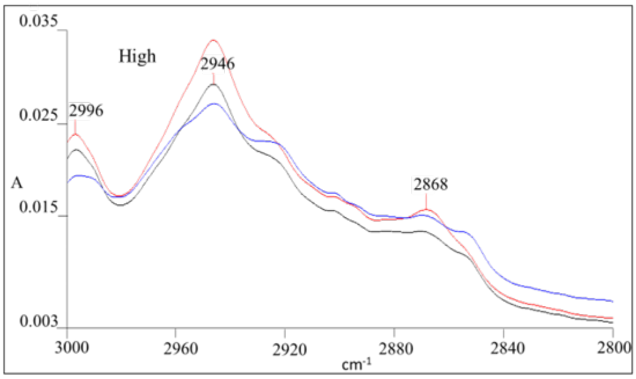

(h)

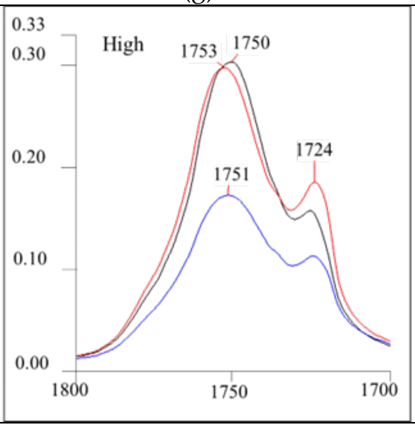

(i)

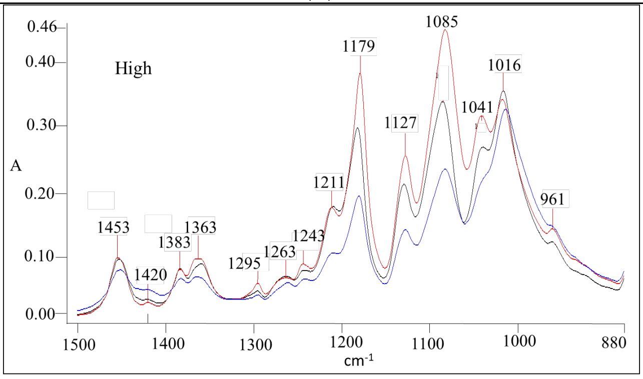

(j)

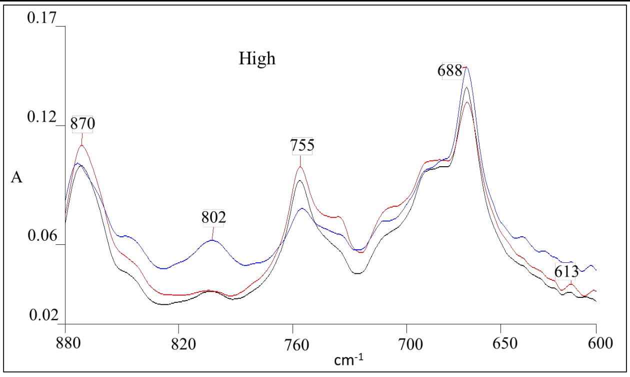

(k)

Figure S3.1. FTIR spectra of bio-hybrids with different PLA-Talc-PCL formulations (**a**) – (**e**): low; (**a**) 3600 cm^{-1} - 3000 cm^{-1}; (**b**) 3000 cm^{-1} - 2800 cm^{-1}; (**c**) 1800 cm^{-1} - 1700 cm^{-1}; (**d**) 1500 cm^{-1} - 880 cm^{-1}; (**e**) 880 cm^{-1} - 600 cm^{-1}); (**f**) – (**k**): high; (**f**) 3690 cm^{-1} - 3660 cm^{-1}; (**g**) 3600 cm^{-1} - 3000 cm^{-1}; (**h**) 3000 cm^{-1} - 2800 cm^{-1}; (**i**) 1800 cm^{-1} - 1700 cm^{-1}; (**j**) 1500 cm^{-1} - 880 cm^{-1}; (**k**) 880 cm^{-1} - 600 cm^{-1}

2. Selected bio-hybrids

Table S3.1. FTIR changes of bio – hybrid with 3.5% PCL (RT 93)

**Bio - hybrid with 3.5% PCL (RT93)**					
**Peaks changes**	**PLA**	**PCL**	**TALC**	**BIO-HYB.**	**TOTAL**
Appear				1	
Disappear	11	15	3	-	29
Shifted with more than 10 cm^{-1}	0	2	3	-	5
Identical or shifted with less than 10 cm^{-1}	10	2	1	-	13
Peaks with H changed by at least 50%	2	2	3	-	7
**Total components peaks**	21	19	7	-	47
**Total peak changes indicating miscibility**:					42
Disappeared or shifted peaks by more than 10 cm^{-1}	11	17	6	-	34
Peaks with H modified by at least 50%	2	2	3	-	7
New peaks	-	-	-	1	1

Table S3.2. FTIR absorptions of bio - hybrid with 3.5% PCL (RT 93)

**Bio - hybrid with 3.5% PCL (RT93)**			
**Peak change type**	**Comp.**	**Wavelength, cm^{-1}**	**Peaks No.**
Disappear	PLA	2928 cm^{-1}; 2900 cm^{-1}; 2880 cm^{-1}; 2860 cm^{-1}; 1452 cm^{-1}; 1381 cm^{-1}; 1359 cm^{-1}; 1310 cm^{-1}; 1266 cm^{-1}; 1210 cm^{-1}; 700 cm^{-1}	11
	PCL	2867 cm-1; 1471 cm^{-1}; 1418 cm^{-1}; 1392 cm^{-1}; 1365 cm^{-1}; 1294 cm^{-1}; 1240 cm^{-1}; 1108 cm^{-1}; 1062 cm^{-1}; 1047cm^{-1}; 961 cm^{-1}; 934 cm^{-1}; 783 cm^{-1}; 731 cm^{-1}; 709 cm^{-1}	15
	Talc	1671 cm^{-1}; 1448 cm^{-1}; 940 cm^{-1}	3
Appear		1014 cm^{-1}	
Shifted with more than 10 cm^{-1}	PLA	-	-
	PCL	1721 cm^{-1} 26 cm^{-1}, H < 23%; 1171 cm^{-1} 10 cm^{-1}, H identic	2
	Talc	1067 cm^{-1} 16 cm^{-1}, H > 280%; 729 cm^{-1} 27 cm^{-1}, H > 30%; 664 cm^{-1} 3 cm^{-1}, H < 74%	3
Identical or shifted with less than 10 cm^{-1}	PLA	2994 cm^{-1} 1 cm^{-1}, H< 13%; 2946 cm^{-1}, H identic; 1747 cm^{-1} 1 cm^{-1}, H < 14%; 1181 cm^{-1}, H < 14%; 1127 cm^{-1} H < 15%; 1083 cm^{-1} H < 21%; 1042 cm^{-1} H > 10% (umar); 955 cm^{-1} H > 525% (umar); 867 cm^{-1} 2 cm^{-1}, H > 60%; 754 cm^{-1} 1 cm^{-1}, H > 19%	10
	PCL	2943 cm^{-1} 3 cm^{-1}, H < 81%; 840 cm^{-1} 9 cm^{-1}, H > 60%	2
	Talc	3674 cm^{-1} 3 cm^{-1}, H < 79%	1

Table S3.3. FTIR changes of bio - hybrid with 3.5% PCL (RT 93) after 2.2 years

Bio - hybrid with 3.5% PCL (RT 93)					
**No.**	**Absorbs, cm-1**	**Assignement***	**Intensity, a.u.**		**↓↑, % after 2.2 years**
			**Initial**	**After 2.2 years**	
1	3677	O-H strtetching vibration from traces of water or surface hydroxyl groups. Uncharacteristic absorption of the component mixture. [1]	0.032	0.005	↓84.38
2	2994	CH3 from PLA and C-H from PCL	0.030	0.014	↓53.33
3	2946	Asymmetric stretching of C-H from PCL and PLA, CH3 from PLA [2]	0.033	0.014	↓57.58
4	1747	Band composed of C=O ester from PLA and C=O from PCL at 1721 cm^{-1}	0.500	0.180	↓64
5	1181	C-O-C from PLA	0.420	0.180	↓57.14
6	1127	Si-O-Mg or Si-O-Si [3]	0.280	0.100	↓64.29
7	1083	C-O from PLA, PCL	0.540	0.200	↓62.96
8	1042	C-O from PLA	0.440	0.110	↓75
9	1014	C-O-C skeleton from PLA [4] and Si-O-Si from talc	0.800	0.200	↓75
10	955	PLA skeleton	0.250	0.100	↓60
11	867	PLA skeleton. Vibration of talc in bio-hybrid. [5]	0.160	0.070	↓56.25
12	849	Merged peak from PLA, PCL, talc [6]	0.080	Disappeared	-
13	754	Merged peak from PLA, PCL	0.130	0.050	↓61.54
14	692	Overlapped vibrations from PCL as catalytical residues at 700 cm^{-1} [7]	0.160	0.070	↓56.25
15	667	C-O and C-H from talc	0.260	0.100	↓61.54

*No. of references in the main text: [62, 63, 64, 65, 66, 67, 68]

[1] Maleki, F. Proliferation and osteogenic differentiation of mesenchymal stem cells on three-dimensional scaffolds made by thermal sintering method. *Chem. pap.* **2021**, 75(2):1-11. DOI: 10.1007/s11696-021-01774-w. [62]

[2] Kemala, T. Preparation and characterization of microspheres based on blend of poly(lactic acid) and poly(ε-caprolactone) with poly(vinyl alcohol) as emulsifier. *Arab. J. Chem.* **2010**, 5(1):103-108. DOI: 10.1016/j.arabjc.2010.08.003. [63]

[3] Kawaguchi, K. FTIR Spectroscopy of NO_3: Observation and Analysis of the 1127 cm-1 Band, International Symposium On Molecular Spectroscopy, 65th Meeting, Ohio State University, 21-25 June **2010.** [64]

[4] Suárez Franco, J.L.; Vázquez-Vázquez, F.C.; Pozos-Guillen, A.J.; Alvarez-Perez, M.A. Influence of diameter of fiber membrane scaffolds on the biocompatibility of hPDL mesenchymal stromal cells. Dent. Mater. J. 2018, 37(3). DOI: 10.4012/dmj.2016-329. [65]

[5] Kondo, Y. Photoactivation of surface peroxides on titanate nanotubes, *Inorg. Chem.* **2025**, 64. DOI:10.1021/acs.inorgchem.5c03709. [66]

[6] Bocchini, S. Comparative study of filler influence on polylactide photooxidation. *EXPRESS Polym. Lett.* **2013**, 7(5):431-442, DOI: 10.3144/expresspolymlett.2013.40. [67]

[7] Xiang, L. Precise synthesis, properties, and structures of cyclic poly(ε-caprolactone)s. *Polym.* **2018**, 10(6):577, DOI: 10.3390/polym10060577. [68]

Table S3.4. FTIR modifications of bio – hybrid with 16% PCL (RT 108)

**Bio - hybrid with 16% PCL (RT 108)**				
**Peaks changes**	**PLA**	**PCL**	**TALC**	**TOTAL**
Disappear	7	11	2	20
Shifted with more than 10 cm^{-1}	0	4	3	7
Identical or shifted with less than 10 cm^{-1}	14	4	2	20
Peaks with H changed by at least 50%	0	4	4	8
**Total components peaks**	21	19	7	47
**Total peak changes indicating miscibility**:<br>Disappeared or shifted peaks by more than 10 cm^{-1}<br>Peaks with H modified by at least 50%	 7<br>0	 15<br>4	 5<br>4	35<br>27<br>8

Table S3.5. FTIR absorptions of bio - hybrid with 16% PCL (RT 108)

**Bio - hybrid with 16% PCL (RT 108)**			
**Peak changes type**	**Comp.**	**Wavelength, cm^{-1}**	**Peaks No.**
Disappear	PLA	2995 cm^{-1}; 2900 cm^{-1}; 2880 cm^{-1}; 1310 cm^{-1}; 1266 cm^{-1}; 955 cm^{-1}; 700 cm^{-1}	7
	PCL	1471 cm^{-1}; 1418 cm^{-1}; 1392 cm^{-1}; 1365 cm^{-1}; 1294 cm^{-1}; 1240 cm^{-1}; 961 cm^{-1}; 934 cm^{-1}; 783 cm^{-1}; 731 cm^{-1}; 709 cm^{-1}	11
	Talc	1671 cm^{-1}; 940 cm^{-1}	2
Shifted with more than 10 cm^{-1}	PLA	-	-
	PCL	1721 cm^{-1} 30 cm^{-1}, H < 26%; 1171 cm^{-1} 10 cm^{-1}, H identic; 1108 cm^{-1} 19 cm^{-1}, H > 56%; 1062 cm^{-1} 24 cm^{-1}, H > 317%	4
	Talc	1067 cm^{-1} 19 cm^{-1}, H > 233%; 729 cm^{-1} 27 cm^{-1}, H > 20%; 664 cm^{-1} 16 cm^{-1}, H < 87%	3
Identical or shifted with less than 10 cm^{-1}	PLA	2946 cm^{-1} H > 31%; 2925 cm^{-1} 3 cm^{-1}, H > 5%; 2851 cm^{-1} H > 11%; 1747 cm^{-1} 4 cm^{-1}, H< 17 %; 1452 cm^{-1} H < 23%; 1381 cm^{-1} 2 cm^{-1}, H<20%; 1359 cm^{-1} H identic; 1210 cm^{-1} H< 15%; 1181 cm^{-1} H <16%; 1127 cm^{-1} 1 cm^{-1}, H< 19%; 1083 cm^{-1} 3 cm^{-1}, H<26%; 1042 cm^{-1} 2 cm^{-1}, H< 18% (umar); 869 cm^{-1} 1 cm^{-1}, H> 40%; 755 cm^{-1} H > 9%	14
	PCL	2943 cm^{-1} 3 cm^{-1}, H < 78%; 2867cm^{-1} H identic; 1047 cm^{-1} 7 cm^{-1}, H > 68%; 840 cm^{-1} 5 cm^{-1}, H identic	4
	Talc	3674 3 cm^{-1}, H < 91%; 1448 cm^{-1} 5 cm^{-1}, H > 100%	2

Table S3.6. FTIR changes of bio - hybrid with 16% PCL (RT 108) after 2.2 years

Bio - hybrid with 16% PCL (RT 108)					
**No.**	**Absorbs, cm^{-1}**	**Assignement***	**Intensity, a.u.**		**↓↑, % after 2.2 years**
			**Initial**	**After 2.2 years**	
1	3677	O-H strtetching vibration from traces of water or surface hydroxyl groups. Uncharacteristic absorption of the components of the mixture. [1]	0.013	0.023	↑76.92
2	2946	Asymmetric stretching of C-H from PCL and PLA, CH_3 from PLA [2]	0.038	0.026	↓31.58
3	2928	CH_2 from PLA and PCL [8]	0.021	0.022	↑4.76
4	2868	CH_2 from PLA and PCL	0.012	0.013	↑8.33
5	2851	CH and CH_2 from PLA and PCL [9]; O-H and Si-O from talc	0.010	0.012	↑20
6	1751	C=O from PLA and PCL	0.480	0.150	↓68.75
7	1725	C=O from PLA and PCL	0.021	0.010	↓52.38
8	1452	Asymmetric bending vibration of CH_3 from PLA	0.100	0.050	↓50
9	1383	CH_3 from PLA (possible crystal [10]),CH_2 from PCL, O-H from talc [11]	0.080	0.050	↓37.5
10	1359	Impurities from all 3 components (Mg or water vibration from talc)	0.100	Disappears	-
11	1364	Impurities	-	0.050	-
12	1210	PLA Conformational changes [12]	0.230	0.160	↓30.43
13	1181	C-O-C from PLA	0.420	0.200	↓52.38
14	1127	Si-O-Mg [3]	0.250	0.170	↓32
15	1083	C-O from PLA, PCL, talc	0.500	0.220	↓56
16	1042	C-O from PLA	0.320	0.210	↓34.38
17	1016	talc (magnesium silicate)	0.470	0.310	↓34.04
18	873	C-COO from PLA [13]	-	0.100	-
19	870	talc in bio-hybrid [5]	0.140	Disappears	-
20	845	C-C from PLA skeleton	0.05	0.06	↑20
21	802	Hydrogen bonds between O-H from talc	0.040	0.050	↑25
22	756	C=O from PCL	0.120	0.080	↓33.33
23	700	PCL, catalytic debris[7]	0.080	0.070	↓12.5
24	680	Si-O-Mg from talc[14]	0.130	0.090	↓30.77
25	660	C-O and C-H from talc	0.180	0.150	↓16.67

*No. of references in the main text [62, 73, 74, 75, 76, 77, 78, 79]

[8] Przybysz-Romatowska, M. *Poly(ε*-caprolactone)/poly(lactic acid) blends compatibilized by peroxide initiators: comparison of two strategies. *Polym.* **2020**, 12(1):228, DOI: 10.3390/polym12010228. [73]

[9] Shojaei, S. Disclosing the role of surface and bulk erosion on the viscoelastic behavior of biodegradable poly(ε-caprolactone)/poly(lactic acid)/hydroxyapatite nanocomposites. *J. Appl. Polym. Sci.* **2018**, 136(10):47151. [74]

[10] Braun, B. Infrared spectroscopic determination of lactide concentration in polylactide: an improved methodology. *Macromol.* **2006**, 39(26), DOI: 10.1021/ma061922a. [75]

[11] Shimadzu Corporation. Application News, Spectrophotometric Analysis. International Marketing Division, No.A422. Printed in Japan 3295-06007-10A-IK. [76]

[12] Liang, J. Polylactide-based chiral particles with enantio-differentiating release ability. *Chem. Eng. J.* **2018**, 344, 262-269. [77]

[13] Tazibt, N. Effect of filler content on the morphology and physical properties of poly(lactic acid)-hydroxyapatite composites. *Mater.* **2023**, 16(2):809. DOI: 10.3390/ma16020809. [78]

[14] Marzbani, P. Surface modification of talc particles with phthalimide: study of composite structure and consequences on physical, mechanical, and optical properties of deinked pulp. *Bioresour.* **2016**, 11(4):8720-8738. DOI: 10.15376/biores.11.4.8720-8738. [79]

Table S3.7. FTIR modifications of bio-hybrid with nucleating agent (RT 103)

**Bio-hybrid with nucleating agent (RT 103)**						
**Peaks changes**	**PLA**	**PCL**	**TALC**	**LAK**	**BIO-HYB.**	**TOTAL**
Appear					1	
Disappear	16	13	3	20	-	52
Shifted with more than 10 cm^{-1}	0	5	3	4	-	12
Identical or shifted with less than 10 cm^{-1}	5	1	1	4	-	11
Peaks with H changed by at least 50%	3	3	3	3	-	12
**Total components peaks**	21	19	7	28	-	75
**Total peaks changes indicating miscibility**:<br>Disappeared or shifted peaks by more than 10 cm^{-1}<br>Peaks with cu H modified by at least 50%<br>New peaks	 16<br>3<br>-	 18<br>3<br>-	 6<br>3<br>-	 24<br>3<br>-	 -<br>-<br>1	77<br>64<br>12<br>1

Table S3.8. FTIR absorptions of bio - hybrid with nucleating agent (RT 103)

**Bio - hybrid with nucleating agent (RT 103)**			
**Peak changes type**	**Comp.**	**Wavelength, cm^{-1}**	**Peaks No.**
Disappear	PLA	2995 cm^{-1}; 2945 cm^{-1}; 2928 cm^{-1}; 2900 cm^{-1}; 2880 cm^{-1}; 2851 cm^{-1}; 1452 cm^{-1}; 1381 cm^{-1};1359 cm^{-1}; 1310 cm^{-1}; 1266 cm^{-1}; 1042 cm^{-1}; 955 cm^{-1}; 867 cm^{-1}; 755 cm^{-1}; 700 cm^{-1}	16
	PCL	2943 cm^{-1}; 2867cm^{-1}; 1471 cm^{-1}; 1418 cm^{-1}; 1392 cm^{-1}; 1365 cm^{-1}; 1294 cm^{-1}; 1240 cm^{-1}; 1062 cm^{-1}; 961 cm^{-1}; 934 cm^{-1}; 840 cm^{-1}; 783 cm^{-1}	13
	Talc	1671 cm^{-1}; 1448 cm$^{-1;}$ 940 cm^{-1}	3
	LAK	3070 cm^{-1}; 2957 cm^{-1}; 1722; cm^{-1} 1601 cm^{-1}; 1441 cm^{-1}; 1428 cm^{-1}; 1322 cm^{-1}; 1219 cm^{-1}; 1196 cm^{-1}; 1098 cm^{-1}; 988 cm^{-1}; 963 cm^{-1}; 932 cm^{-1}; 917 cm^{-1}; 887 cm^{-1}; 875 cm^{-1}; 851 cm^{-1}; 782 cm^{-1}; 764 cm^{-1}; 623 cm^{-1}	20
Appear		1014 cm^{-1}	
Shifted by more than 10 cm^{-1}	PLA	-	-
	PCL	1721 cm^{-1} 29 cm^{-1}, H < 60%; 1171 cm^{-1} 10 cm^{-1}, H < 40%; 1108 cm^{-1} 10 cm^{-1}, H > 13%; 1047 cm^{-1} 38 cm^{-1}, H > 53%; 731 cm^{-1} 25 cm^{-1}, H < 53%	5
	Talc	1067 cm^{-1} 18 cm^{-1}, H > 94%; 729 cm^{-1} 27 cm^{-1}, H < 10%; 664 cm^{-1} 21 cm^{-1}, H < 88%	3
	LAK	1734 cm^{-1} 16 cm^{-1}, H identic; 1139 cm^{-1} 42cm^{-1}, H < 19%; 1117 cm^{-1} 11 cm^{-1}, H < 18%; 1048 cm^{-1} 37cm-1, H < 12%;	4
Identical or shifted with less than 10 cm^{-1}	PLA	1747 cm^{-1} 3 cm^{-1}, H < 55 %; 1210 cm^{-1} H < 44%; 1181 cm^{-1} H < 51%; 1127 cm^{-1} 1 cm^{-1}, H < 45%; 1083 cm^{-1} 2 cm^{-1}, H < 57%	5
	PCL	709 cm^{-1} 7 cm^{-1}, H <38%	1
	Talc	3674 cm^{-1} 2 cm^{-1}, H < 91%	1
	LAK	1210 cm^{-1} H < 67%; 751 cm^{-1} 5 cm^{-1}, H < 72%; 720 cm^{-1} 4 cm^{-1}, H < 64%; 676 cm^{-1} 9 cm^{-1}, H < 29%	4

Table S3.9. FTIR changes of bio - hybrid with nucleating agent (RT 103) after 2.2 years

**Bio - hybrid with nucleating agent (RT 103)**					
**No.**	**Absorbs, cm^{-1}**	**Assignement**	**Intensity, a.u.**		**↑↓, % after 2.2 years**
			**Initial**	**After 2.2 years**	
1	3676	3674 cm^{-1} from talc, shifted with 2 cm^{-1}	0.014	0.014	0
2	1750	Peak merged from 1747 from PLA from 1747 cm^{-1} (shifted with 3 cm^{-1}), 1721 cm^{-1} from PCL (shifted with 29 cm^{-1}), 1734 cm^{-1} from nucleating agent shifted with 16 cm^{-1}	0.26	0.19	↓ 26.92
3	1210	Peak merged from PLA shoulder from 1210 cm^{-1} and nucleating agent peak from 1210 cm^{-1}	0.15	0.11	↓26.67
4	1181	Peak merged from PLA from 1181 cm^{-1} and from PCL from 1171 cm^{-1} shifted with 10 cm^{-1}, and possibly also with the one from nucleating agent from 1139 cm^{-1} shifted with 42 cm^{-1}	0.25	0.20	↓20
5	1128	Peak merged from PLA from 1127 cm^{-1}, with the one from PCL from 1108 cm^{-1} shifted with 20 cm^{-1} and the nucleating agent peak from 1117 cm^{-1} shifted with 11 cm^{-1}	0.18	0.15	↓16.67
6	1085	Peak merged from 1083 cm^{-1} from PLA, with the one from talc from 1067 cm^{-1} shifted with 18 cm^{-1}, and the peak from 1047 cm^{-1} from PCL, shifted with 38 cm^{-1} and the nucleating agent peak from 1048 cm^{-1}, shifted with 37 cm^{-1}	0.29	0.25	↓13.79
7	1014	New Peak	0.31	0.30	↓3.23
8	756	Peak merged from 755 cm^{-1} from PCL, 729 cm^{-1} from talc, shifted with 26 cm^{-1}, 731 cm^{-1} from PCL shifted with 25 cm^{-1} and the nucleating agent peak from 751 cm^{-1}	0.09	0.07	↓22.22
9	716	Peak merged from PCL peak from 709 cm^{-1}, shifted with 7 cm^{-1}, and the nucleating agent peak from 720 cm^{-1}	0.05	0.05	↓0
10	685	Peak merged from talc peak from 664 cm^{-1}, shifted with 21 cm^{-1} and the nucleating agent peak from 676 cm^{-1}, shifted with 9 cm^{-1}	0.12	0.11	↓8.33
