# Supplementary material for: The Influence of Miscibility of Some PLA-Based Bio-Hybrids Designed for 3D Printing and Medium-Life Applications on Their Physical Aging and Thermodynamic Stability
Source: Polymers (Basel). 2025 Dec 25;18(1):61. doi: 10.3390/polym18010061 (PMC12788091; doi:10.3390/polym18010061)
Supplement: Supplementary file 1 [file polymers-18-00061-s001.zip › Supplementary Material 4 (S4).pdf]

Supplementary material 4 (S4)

Table S4.1. XRD data and crystallinity of bio - hybrids (with 3.5% PCL, 16% PCL and with nucleating agent) initially and after 2.2 years

**Bio - hybrid**		**Diffractions (angle, 2θ° and intensity, counts/s)**	**Total peaks no.**	**Crystallinity %**
Bio - hybrid with 3.5% PCL (RT 93)	Initial	15° - 188000 cps; 21° - 42000 cps; 32° - 47000 cps; 53° - 14000 cps; 63° - 8000 cps; 75° - 8000 cps;	6	29.7
	After 2.2 years	15° - 178000 cps; 21° -18000 cps; 23° - 36000 cps; 32° - 125000 cps; 43° - 7000 cps; 53° - 18000 cps; 63° - 10000 cps; 75° - 10000 cps	8	23.4
Bio – hybrid with 16% PCL (RT 108)	Initial	10° - 12000 cps; 13° - 55000 cps; 16° - 10000 cps; 22° - 18000 cps; 23° - 20000 cps; 26° - 12000 cps; 33° - 58000 cps; 54° -11000 cps; 76° - 13000 cps	9	5 (1.1% at 57.5°C; 3.9% at 155.8°C)
	After 2.2 years	11° - 13000 cps; 13° - 130000 cps; 16° - 12000 cps; 22° - 21000 cps; 23° - 29000 cps; 26° - 15000 cps; 30° -12000 cps; 33° -82000 cps; 54° - 17000 cps; 64° - 15000 cps; 76° - 14000 cps	11	6.8 (2% at 57.3°C; 4.8% at 155.3°C)
Bio – hybrid with nucleating agent (RT 103)	Initial	10° - 12000 cps; 13° - 182000 cps; 17° - 12000 cps; 21° - 23000 cps; 23° - 28000 cps; 30° - 11000 cps; 34° - 100000 cps; 53° -18000 cps; 63° - 12000 cps;	9	19.3
	After 2.2 years	10° - 18000 cps; 13° - 132000 cps; 17° - 12000 cps; 21° - 19000 cps; 23° - 23000 cps; 30° - 11000 cps; 34° - 90000 cps; 53° -18000 cps; 63° - 12000 cps; 75° - 10000 cps	10	19.1
