# Supplementary material for: The Influence of Miscibility of Some PLA-Based Bio-Hybrids Designed for 3D Printing and Medium-Life Applications on Their Physical Aging and Thermodynamic Stability
Source: Polymers (Basel). 2025 Dec 25;18(1):61. doi: 10.3390/polym18010061 (PMC12788091; doi:10.3390/polym18010061)
Supplement: Supplementary file 1 [file polymers-18-00061-s001.zip › Supplementary Material 5 (S5).pdf]

# Supplementary Material 5 (S5)

**Bio - hybrid with 3.5% PCL (RT 93) after 2.2 years**

**2000X** **2000X** **5000X**

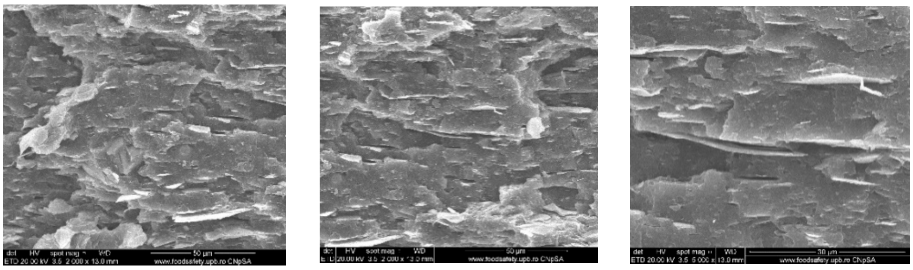

**10000X** **1000X** **500X**

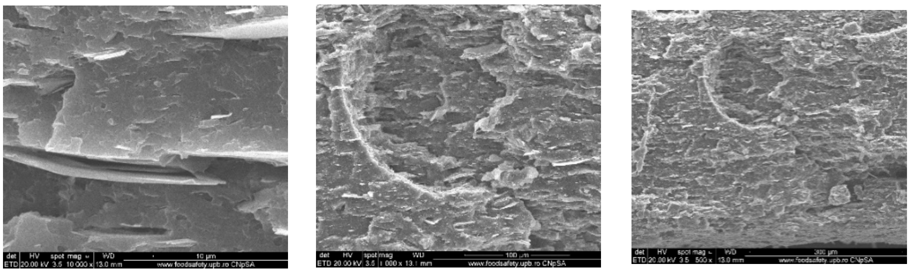

**Figure S5.1**. SEM morphology of bio - hybrid with 3.5% PCL (RT 93) after 2.2 years

**Bio - hybrid with 16% PCL (RT 108) inittial**

**1000X** **2000X** **4000X** **10000X**

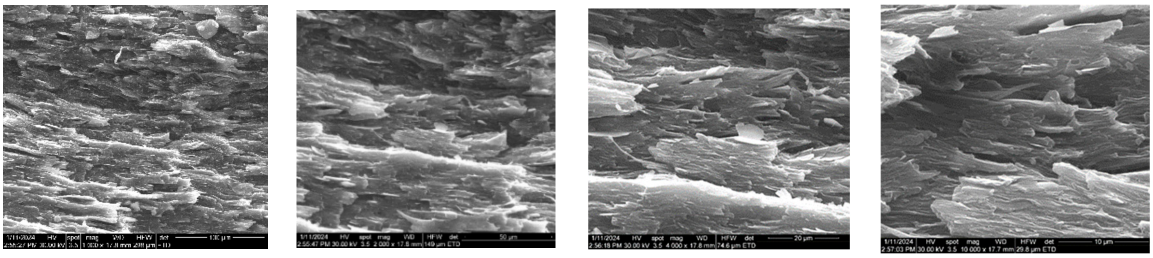

**Bio - hybrid with 16% PCL (RT 108), after 2.2 years**

**1000X** **2000X** **4000X** **10000X**

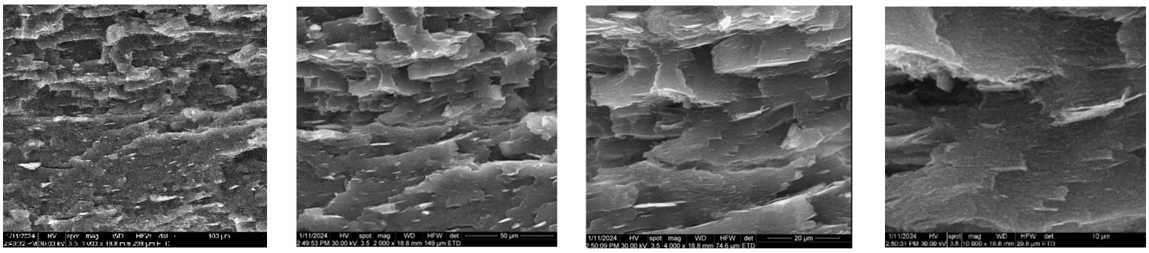

**Figure S5.2**. SEM morphology of bio - hybrid with 16% PCL (RT 108) initially and after 2.2 years

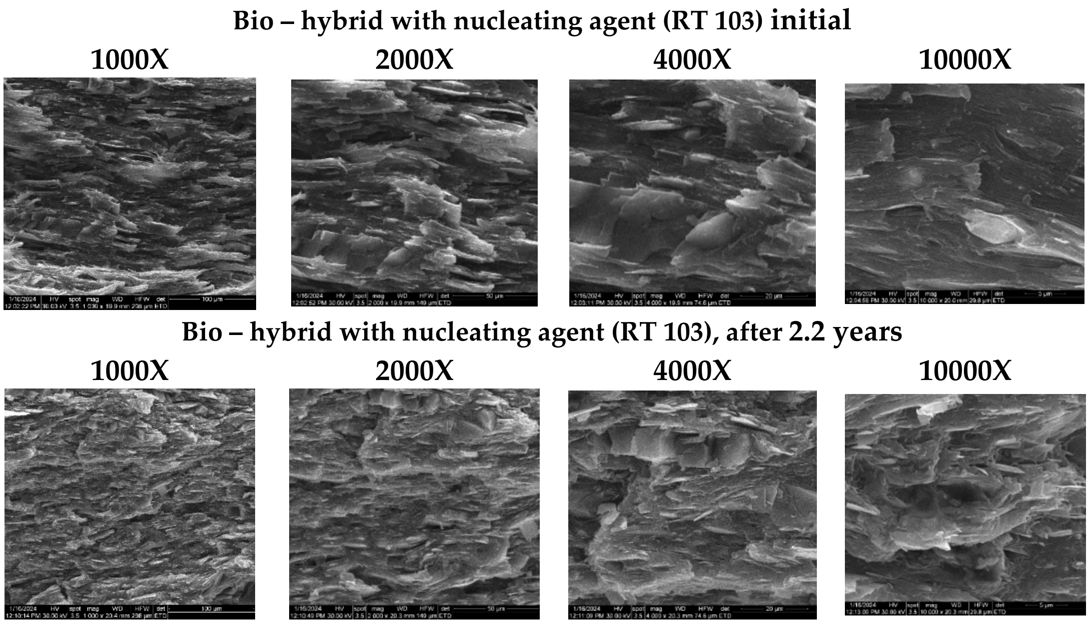


**Figure S5.3**. SEM morphology of bio - hybrid with nucleating agent (RT 103) initial and after 2.2 years
