# Supplementary material for: The Influence of Miscibility of Some PLA-Based Bio-Hybrids Designed for 3D Printing and Medium-Life Applications on Their Physical Aging and Thermodynamic Stability
Source: Polymers (Basel). 2025 Dec 25;18(1):61. doi: 10.3390/polym18010061 (PMC12788091; doi:10.3390/polym18010061)
Supplement: Supplementary file 1 [file polymers-18-00061-s001.zip › Supplementary Material 6 (S6).pdf]

Supplementary Material 6 (S6)

Table S6.1. Physical properties of components and bio – hybrids (initial and after 2.2 years)

**Property**	**Initial**	**After 2.2 years**	**$\Delta_{2.2,2.2-i}$**
**Density**, g/cm^3; Hardness, ^{0}Sh A			
**A. Components**			
Neat PLA	1.24		
PCL	1.08-1.12		
Talc	2.7		
Lak 301	1.70		
**B. Bio-hybrids**			
Bio - hybrid with 3.5% PCL (RT 93)			
Density, g/cm^3	1.355	1.411	0.056↑
Hardness, ^{0}Sh A	94	95	1↓
Bio – hybrid with 16% PCL (RT 108)			
Density, g/cm^3	1.289	1.299	0.010 ≈
Hardness, ^{0}Sh A	91	91	0
Bio – hybrid with nucleating agent (RT 103)			
Density, g/cm^3	1.510	1.532	0.022↑
Hardness, ^{0}Sh A	95	95	0
