# Supplementary material for: The Influence of Miscibility of Some PLA-Based Bio-Hybrids Designed for 3D Printing and Medium-Life Applications on Their Physical Aging and Thermodynamic Stability
Source: Polymers (Basel). 2025 Dec 25;18(1):61. doi: 10.3390/polym18010061 (PMC12788091; doi:10.3390/polym18010061)
Supplement: Supplementary file 1 [file polymers-18-00061-s001.zip › Supplementary Material 7 (S7).pdf]

Supplementary Material 7 (S7)

Table S7.1. Mechanical behavior after 2.2 years of bio – hybrids with 3.5% PCL, 16% PCL and with nucleating agent

**Estimated mechanical behavior after 2.2 years**			
**Test according to Subchapter 2.4.**	**With 3.5% PCL (RT 93)**	**With 16% PCL (RT 108)**	**With nucleating agent (RT 103)**
Tensile strength	Yes	Yes	Yes
Flexural strength	Yes	Yes	Yes
Impact resistance	Yes	Yes	Yes
